# Dependence, withdrawal and rebound of CNS drugs: an update and regulatory considerations for new drugs development

**DOI:** 10.1093/braincomms/fcz025

**Published:** 2019-10-16

**Authors:** Alicja Lerner, Michael Klein

**Affiliations:** 1 Center for Drug Evaluation and Research, Food and Drug Administration, Silver Spring, MD 20993-0002, USA; 2 Controlled Substance Scientific Solutions LLC, 4601 North Park Avenue #506, Chevy Chase, MD 20815-4572, USA

**Keywords:** dependence, withdrawal syndromes, rebound, human dependence evaluation

## Abstract

The purpose of this article is to describe dependence and withdrawal phenomena related to CNS drugs discontinuation and to clarify issues related to the evaluation of clinical drug withdrawal and rebound as they relate to safety in new drug development. The article presents current understanding and definitions of drug dependence and withdrawal which are also relevant and important features of addiction, though not the same. Addiction, called substance use disorder in DSM-5, affects an individual’s brain and behaviour, represents uncontrollable drug abuse and inability to stop taking a drug regardless of the harm it causes. Characteristic withdrawal syndromes following abrupt discontinuation of CNS-active drugs from numerous drug classes are described. These include drugs both scheduled and non-scheduled in the Controlled Substances Act, which categorizes drugs in five schedules based on their relative abuse potentials and dependence liabilities and for regulatory purposes. Schedules 1 and 2 contain drugs identified as those with the highest abuse potential and strictest regulations. Less recognized aspects of drug withdrawal, such as rebound and protracted withdrawal syndromes for several drug classes are also addressed. Part I presents relevant definitions and describes clinical withdrawal and dependence phenomena. Part II reviews known withdrawal syndromes for the different drug classes, Part III describes rebound and Part IV describes protracted withdrawal syndromes. To our knowledge, this is the first compilation of withdrawal syndromes for CNS drugs. Part V provides details of evaluation of dependence and withdrawal in the clinical trials for CNS drugs, which includes general design recommendations, and several tools, such as withdrawal questionnaires and multiple scales that are helpful in the systematic evaluation of withdrawal. The limitations of different aspects of this method of dependence and withdrawal evaluation are also discussed.

## Teaser

This manuscript introduces the concepts of dependence, withdrawal and rebound for CNS-active drugs and provides a comprehensive review of multiple currently known withdrawal syndromes for scheduled and non-scheduled drugs. The manuscript also describes details of evaluation of dependence and withdrawal in clinical trials for drugs that are being developed.

## Graphical Abstract

**Graphical Abstract:**
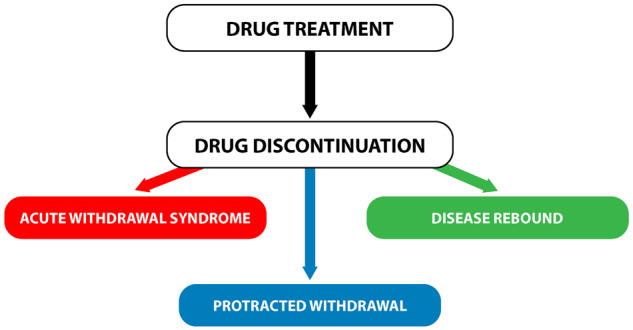

## Part I: Introduction, definitions and general considerations of drug dependence, withdrawal and rebound 

### Introduction

In the United States, the significance of drug dependence, especially as it relates to addiction, came to the foreground and captured public attention only in recent decades, following epidemics of drug abuse, dependence and addiction, sweeping the country, resulting in staggering numbers of deaths. Most cases are related not only to opioid drugs, but also to benzodiazepines (BZ), stimulants and street drugs, including heroin, highly potent fentanyl and its analogues, cathinone-like hallucinogens and dissociative substances, and stimulants similar in action to cocaine and methamphetamine (Dowell *et al.*, 2017; Scholl *et al.*, 2018; Colon-Berezin *et al.*, 2019; Kariisa *et al.*, 2019).

Serious and potentially life-threatening adverse events related to drug withdrawal and dependence may result also from other drug classes generally not associated with abuse and not scheduled under the Controlled Substances Act. Withdrawal of antidepressants and other CNS drugs such as BZ and stimulants may lead to dangerous, even life-threatening, withdrawal symptoms and lead to significant public health concerns, such as suicidality (Risse *et al.*, 1990; Valuck *et al.*, 2009; APA, 2013; Fava *et al.*, 2015).

A widespread misunderstanding regarding dependence and withdrawal is that both need to be related to drug abuse and addiction. Also, in general, the occurrence of dependence and withdrawal may be underestimated, and their significance underappreciated, particularly for drugs not known for abuse potential and which are not scheduled substances in the Controlled Substances Act (Newman *et al.*, 2009; Rabinak and Nirenberg, 2010; Reidenberg, 2011; Hudak *et al.*, 2012; Chouinard and Chouinard, 2015; Fava *et al.*, 2015).

Thus, this article has two main goals, one is to provide brief condensed summaries of withdrawal syndromes for the majority of the CNS-active drug groups which are of interest to prescribers, patients and also clinical scientists, this addresses a gap of knowledge usually not readily available in the drug labels.

The second goal is to emphasize the importance of evaluating dependence and withdrawal for all CNS-active drugs and to provide standardized advice how to assess symptoms of drug withdrawal. The information obtained will be used to provide safety data for warnings on drug labels and to inform patients and physicians about possible dependence, withdrawal and rebound related to the drug—and their consequences.

### Definitions of dependence and withdrawal

The phrase ‘drug dependence and withdrawal’ most often and traditionally relates to substance abuse. However, at other times it can relate to a broader concept of ‘dependence and withdrawal’, which potentially may encompass all drug classes, because it may be viewed as a function of an organism’s adaptation to the presence of a drug in the body. In this study, we will discuss both approaches.

In the DSM-5 (Diagnostic and Statistical Manual of Mental Disorders, Fifth Edition; APA, 2013) definitions of drug dependence and withdrawal related to substance use disorder are described in the ‘Substance-Related and Addictive Disorders’ section, and are further defined for drugs and substances known to be related to substance use disorders. These substances include opioids, sedatives, hypnotics, anxiolytics, stimulants, cannabis, caffeine, alcohol and tobacco. Of these substances, cannabis is listed in Schedule 1 of the Controlled Substances Act, many opioids and stimulants are listed in Schedule 2; sedatives, hypnotics and anxiolytics are listed in Schedule 3 or 4, whereas caffeine, alcohol and tobacco are not listed in the Controlled Substances Act (United States Congress, 1970).

However, for drugs, in particular CNS active, which are being developed and evaluated by FDA, we will be using another definition, more appropriate for this function, namely, part (i) of the dependence definition cited by the American Society of Addiction Medicine (ASAM). The recent ASAM definitions of dependence, addiction, tolerance and withdrawal gained support by the American Academy of Pain Medicine and the American Pain Society in 2001, and have since then been updated (Ries *et al.*, 2014).

- Physical dependence—used in three different ways: (i) physical dependence is a state of adaptation that is manifested by a drug class-specific withdrawal syndrome that can be produced by abrupt cessation, rapid dose reduction, reducing blood level of the drug and/or administration of an antagonist; (ii) psychological dependence is a subjective sense of need for a specific psychoactive substance, either for its positive effects or to avoid negative effects associated with its abstinence; and (iii) one category of psychoactive substance use disorder in previous editions of the Diagnostic and Statistical Manual of Mental Disorders, but not in the DSM-5, published in 2013.
- Addiction is characterized by inability to consistently abstain, impairment in behavioural control, craving, diminished recognition of significant problems with one’s behaviours and interpersonal relationships, and a dysfunctional emotional response. Like other chronic diseases, addiction often involves cycles of relapse and remission. Without treatment or engagement in recovery activities, addiction is progressive and can result in disability or premature death.
- Tolerance—a state of adaptation in which exposure to a drug over time results in diminution of one or more of the drug’s physiologic effects.
- Withdrawal syndrome—the onset of a predictable constellation of signs and symptoms following the abrupt discontinuation of, or rapid reduction in, the dose of a psychoactive substance.

These definitions are important for distinguishing between dependence and addiction or substance use disorder.

To further clarify the concept of dependence it can be said that an individual can be addicted to the drug of abuse, or merely develop dependence to the drug of abuse without being addicted. For drugs not associated with abuse potential, an individual may still develop dependence; but again, this would not be classified as an addiction.

The phenomena of dependence are related to withdrawal, tolerance and rebound. Ashton (1991) described the relation of withdrawal, rebound and tolerance using the example of BZ. The excerpt below is somewhat lengthy for the citation, but it describes withdrawal phenomena, their sequence and physiological background so well that the authors decided to provide it here in full.

Any chronically used drug gradually engenders a series of homeostatic responses which tend to restore normal function despite the presence of the drug. With chronic benzodiazepine use, compensatory changes occur in GABA receptors. Such changes consist of decreased sensitivity of these receptors to GABA, probably as a result of alterations in affinity state and decreased density (Cowen and Nutt, 1982; Nutt and Malizia, 2001).

In addition, there are changes in the secondary systems controlled by GABA, so that the output of excitatory neurotransmitters tends to be restored, and/or the sensitivity of their receptors increases. The whole complex of primary and secondary changes eventually results in benzodiazepine *tolerance*.

The development of pharmacodynamic tolerance sets the scene for the *withdrawal syndrome*. Cessation of the drug exposes all the adaptations which have accrued to counteract its presence, releasing a *rebound* of unopposed activity involving many neurotransmitters and their receptors and many brain systems. Clinically this state is manifested as the *withdrawal syndrome*, consisting of effects that are largely the opposite of these originally induced by the drug.

Evaluation of dependence and withdrawal is an integral part of FDA’s evaluation of new drugs.

The development of dependence is a general feature of drug effects on the human body which frequently cannot be predicted *a priori*. Thus, it is important to evaluate thoroughly new drugs for development of dependence, withdrawal and rebound symptoms, as well for development of abuse potential (FDA Abuse Guidance 2017; FDA and Center for Drug Evaluation and Research, 2017).

The evaluation of human dependence and withdrawal is important for regulatory reasons, as it relates to 21U.S.C. 811 and 812 (United States Code, Title 21—FOOD AND DRUGS; U.S. Government, 2006): a drug that is a controlled substance, needs to be monitored for abuse and misuse and new data that relate to abuse and dependence liability resulting from abuse needs to be assessed.

The assessment of dependence and withdrawal is also necessary as a potential warning for physicians and patients and should be provided in the drug label to inform if the drug can be abruptly withdrawn at the end of treatment or must be slowly tapered to avoid potentially dangerous even life-threatening adverse events after abrupt withdrawal. It is also important to inform subjects abusing the drug about health consequences of the development of dependence and of drug withdrawal.

### Drug dependence and withdrawal

We present here withdrawal-related phenomena which can be identified for many different drug groups; this list is based largely on a description of clinical aspects of withdrawal emerging after discontinuation of selective serotonin reuptake inhibitors (SSRIs; Chouinard and Chouinard, 2015). These withdrawal phenomena include:

- *New symptoms (acute withdrawal symptoms)*: Newly emerging signs and symptoms which occur almost immediately after abrupt drug discontinuation or sometimes even after the dose decrease. These symptoms are related to the disruption of neuroregulatory changes (neuroadaptation) established during drug administration.
- *Rebound*: Recurrence of symptoms of the treated disorder in patients, more severe than before the treatment.
- *Protracted withdrawal syndrome*: Usually appears long past the timeframe of acute withdrawal symptoms, may last for weeks and months, and sometimes may present as a newly emerging disorder.
- *Relapse*. Return of signs and symptoms of the disease after a remission due to natural causes or termination of treatment; it occurs as a phenomenon in the natural history of the disorder. Relapse is not an aspect of dependence; however, it is mentioned here because it occurs during the timeframe of acute withdrawal and needs to be differentiated from the withdrawal.
- *Symptoms of delayed drug toxicity*: May be over-imposed on acute withdrawal symptoms and sometimes are difficult to differentiate from them.

### Factors influencing formation of drug dependence

Several studies were performed to evaluate the influence of various factors on the development of dependence. A general consensus is that: (i) timing and rate of exposure(s); (ii) dose; and (iii) drug potency, critically impact the formation of dependence, where with a longer timeframe of use, higher dose and higher drug potency, the likelihood of dependence formation increases (MacKinnon and Parker, 1982; Owen and Tyrer, 1983; Roy-Byrne *et al.*, 1989; Rickels *et al.*, 1990; Busto and Sellers, 1991; Noyes *et al.*, 1991; Chouinard, 2004; Kan *et al.*, 2004).

The neurotransmitter system affected by the drug is also an important variable. Opiate, GABA-ergic and dopaminergic systems tend to be more frequently linked to the development of dependence. However, the impact of other factors, such as gender and age, is less known.

#### The length of exposure necessary for development of dependence

Probably the first studies examining the relationship between time of drug exposure, dose and formation of dependence and withdrawal were performed in psychiatric patients by Hollister *et al.* (1961, 1963) using the BZ, chlordiazepoxide and diazepam. Since then, it has been widely recognized that formation of physical dependence resulting in a clinically significant withdrawal syndrome can occur within 6 weeks of BZ administration, especially if sufficiently high doses (two to three times the therapeutic dose) are used. MacKinnon and Parker (1982) reviewed the literature on this topic and found that withdrawal may occur even after only 4–8 weeks of drug exposure; however, in these cases frequently excessively high doses were used.

Kales *et al.* (1987) observed that after only 7 days of administration of alprazolam (1 mg) insomnia patients develop tolerance and drug lost ∼40% of efficacy, additionally abrupt withdrawal caused rebound insomnia.

Roy-Byrne *et al.* (1989) compared the withdrawal effects of alprazolam and diazepam in patients with panic disorder after 6 weeks of drug administration. After an initial taper, abrupt discontinuation of drug treatment resulted in relapse and rebound in the patients taking alprazolam and diazepam, with higher prevalence of these symptoms for alprazolam.

Dependence even after therapeutic exposure to opioids such as oxycodone develops even more rapidly, within 2–3 weeks as described by Wakim (2012), Pasero and McCaffery (2010) and Dowell *et al.* (2016) in Oxycontin label September 2018 and Roxybond label April 2017.

These studies would suggest that dependence formation after administration of opioids can occur within 2–3 weeks using therapeutic doses and for BZ can occur within 6 weeks using therapeutic doses of BZ and in 4 weeks with supratherapeutic doses.

### Sex and age differences—a possible factor in severity of withdrawal

#### Animal withdrawal studies, sex and age impact

Non-clinical withdrawal studies in rodents showed that there are sex and age differences in the severity of symptoms and duration of withdrawal as described in Box 1.

Box 1 Animal withdrawal studies, sex and age impact

*Opioids*

Cicero *et al*. (2002) found that there were sex-related differences in the severity of an opioid withdrawal syndrome after cessation of chronic morphine administration. After stopping the chronic morphine injections or morphine pellet implantation, male rats experienced more severe spontaneous withdrawal syndrome than female rats, consisting of wet-dog shakes, diarrhoea and prominent body weight loss; also, the duration of the withdrawal syndrome was longer in male rats.

In a more recent study, Bobzean *et al.* (2019) also observed that male morphine-dependent rats expressed more severe symptoms (wet-dog shakes, writhing, global withdrawal score) during the early phases of spontaneous withdrawal compared with females.

*Benzodiazepines*

Pesce *et al.* (1994) reported sex differences in withdrawal symptoms of intact and castrated mice that were first treated with subcutaneously implanted pellet of diazepam and then underwent precipitated withdrawal with flumazenil injection. Both sexes experienced seizures and jerks; however, seizures were more pronounced in females. Castration significantly increased the incidence of seizures in male mice.

Sloan *et al.* (2000) observed the sex differences in the number in seizures, respiratory rate and vocalization in rats which first had implanted diazepam and then underwent withdrawal precipitated with either with PK11195, a peripheral BZ antagonist, and/or flumazenil. Flumazenil induced higher withdrawal scores in females, also female rats vocalized significantly more than male rats; however, flumazenil tended to produce more tachypnoea in male than in female rats.

*Cannabis*

Marusich *et al.* (2014) studied impact of gonadal hormones on Δ9-tetrahydrocannabinol (THC) dependence in gonadectomized rats. Rimonabant, selective cannabinoid 1 receptor blocker, precipitated withdrawal in rats with increased forepaw tremors, licking, and increased startle amplitude. However, testosterone treatment of gonadectomized males decreased withdrawal-induced licking, whereas oestradiol and progesterone treatment of gonadectomized females increased withdrawal-induced chewing, and progesterone increased withdrawal-induced sniffing.

Harte-Hargrove and Dow-Edwards (2012) who studied the sex differences in adolescent THC-dependent rats after the spontaneous THC withdrawal showed increased anxiety-like behaviour in females and decreased anxiety-like behaviour in males early in withdrawal, also female rats showed greater locomotor depression than males.

*Nicotine*

Torres *et al.* (2013) reported that during nicotine withdrawal, adult female rats displayed higher levels of anxiety-like behaviour than adult males. However, adolescents displayed less nicotine withdrawal than adults, but adolescent males displayed some increase in anxiety-like behaviour.

Hamilton *et al.* (2010) observed more severe withdrawal behaviours in adolescent male Long-Evans than females. However, these sex differences were not seen in the adolescent Sprague Dawley male and female rats, both sexes had significant withdrawal behaviours.

#### Sex and age differences in humans during drugs withdrawal

There are limited human data available regarding difference in severity of withdrawal symptoms by sex; some studies discuss this topic for different pharmacological groups (Agrawal *et al.*, 2008; Copersino *et al.*, 2010; Chen *et al.*, 2014; Chartier *et al.*, 2015; Herrmann *et al.*, 2015; Cuttler *et al.*, 2016; Rungnirundorn *et al.*, 2017).

##### Opioids

Back *et al.* (2011), studied demographic characteristics and substance use parameters among opioid-dependent men and women participating in a multisite effectiveness trial.

Withdrawal symptoms in this population were assessed using the Opiate Withdrawal Symptoms and Craving (COWS), Adjective Rating Scale for Withdrawal (ARSW) and Visual Analogue Scale (VAS). The results showed that gender difference examined with COWS was small (effect size = 0.12), also no statistically significant gender-related differences in withdrawal symptoms were revealed with the ARSW or VAS. Subjective craving as measured with VAS was significantly higher in women than men.

However, further research studies with humans, males and females, are needed to investigate sex and gender variables that influence the various conditions affected by opioids, including treating pain, occurrence of adverse events and development of addiction (Koons *et al.*, 2018).

##### Cannabis

Agrawal *et al.* (2008) examined symptoms of cannabis withdrawal in subjects who reported its use during the past 12 months to the National Epidemiologic Survey on Alcohol and Related Conditions (NESARC). It was noted that nausea was more frequently reported by women (3.2% versus 1.7%) and goose bumps and pupil dilation more frequently reported by men (2.2% versus 4.6%) at *P* = 0.02.

Copersino *et al.* (2010), in a study that evaluated sociodemographic characteristics of the cannabis withdrawal experience, reported that women were more likely than men to report physical withdrawal symptoms. Also, during cannabis withdrawal women reported more symptoms of upset stomach but significantly fewer instances of craving or increased sex drive compared with men.

Herrmann *et al.* (2015) reported on sex differences during cannabis withdrawal which were assessed with the Marijuana Withdrawal Checklist (MWC), then converted into a composite Withdrawal Discomfort Scale (WDS) score. Women had significantly higher composite WDS scores than men as well as higher scores than men on six individual symptoms in two domains: mood symptoms that included irritability, restlessness, increased anger and violent outbursts; and gastrointestinal symptoms, that included nausea and stomach pain.

Cuttler *et al.* (2016) based on the online survey that assessed the cannabis users’ practices and experiences found that during the withdrawal period men more often than women reported insomnia and vivid dreams, whereas women more often reported nausea and anxiety. Also, significantly more women (44.9%) than men (36.7%) did not experience withdrawal symptoms.

##### Stimulants

Chartier *et al.* (2015) examined sex-specific factors associated with severity of stimulant withdrawal in a population of abusing or dependent subjects undergoing treatment. The results of the study that used Stimulant Selective Severity Assessment (SSSA), showed that women reported greater severity of stimulant withdrawal, namely 13.78 (SD 11.9) than did men 10.44 (SD 11.1). This difference was seen in anxiety-related withdrawal symptoms (anxiety, tension, attention and irritability) which were more frequent in women.

Rungnirundorn *et al.* (2017) evaluated withdrawal symptoms in both genders of methamphetamine users. Females were more likely than males to be methamphetamine dependent (79% versus 60%), and to experience withdrawal (65.3% versus 48.9%). Also, some withdrawal symptoms were more frequent in women versus men, including hypersomnia (77.2% versus 64.8%), fatigue (77.5% versus 70.3%).

##### Ketamine

Chen *et al.* (2014) analysed sex differences in subjective withdrawal symptoms associated with ketamine use. Results of the study showed that female ketamine users, compared with male users, reported meaningful difference in withdrawal symptoms, such as anxiety (23.4% versus 17.4%), dysphoria (24.1% versus 15.9%) and cravings (23% versus 17.9%).

##### Nicotine

Foulkner *et al.* (2018) examined in young adult smokers impact of varied yields of nicotine on sex differences in craving, withdrawal and affect. Women reported greater psychological withdrawal, greater sedation and trend towards greater craving than men during abstinence.

### Age as a factor in dependence formation and severity of withdrawal syndrome

There are some data available describing impact of age on dependence formation, mainly in relation to opioids and BZ. It was noted that in children undergoing treatment with opioids, physical dependence may develop in as little as 2–3 days of continuous opioid therapy (Fisher *et al.*, 2013). Also, it seems that signs of opioid withdrawal are more severe in older children than in younger children and infants; thus, adolescents and children more than 7 years old are particularly at risk for increased severity of withdrawal (Anand and Ingraham, 1996).

Regarding BZ withdrawal in children, in addition to classic BZ withdrawal symptoms, children may show visual hallucinations, agitation, facial grimacing, aerophagia, myoclonus, ataxia, choreoathetoid movements and generalized seizures (Anand and Ingraham, 1996). Dependence and resulting withdrawal syndrome may develop in only 7–17 days, although doses of midazolam used in the cases described were high (Sury *et al.*, 1989).

Also, older adults (aged >50 years) seem to develop somewhat different withdrawal symptoms than do younger people after BZ discontinuation. These include emergence of confusion and disorientation, sometimes with hallucinations in older people instead of anxiety, insomnia and perceptual changes noted in younger people (Kruse, 1990; Simoni-Wastila and Yang, 2006). Also, it was observed that catatonia after BZ withdrawal occurs mainly in older adults (Rosebush and Mazurek, 1996; Lebin and Cerimele, 2017).

### Five Half-life times rule—issues and misunderstandings

**Figure 1:**
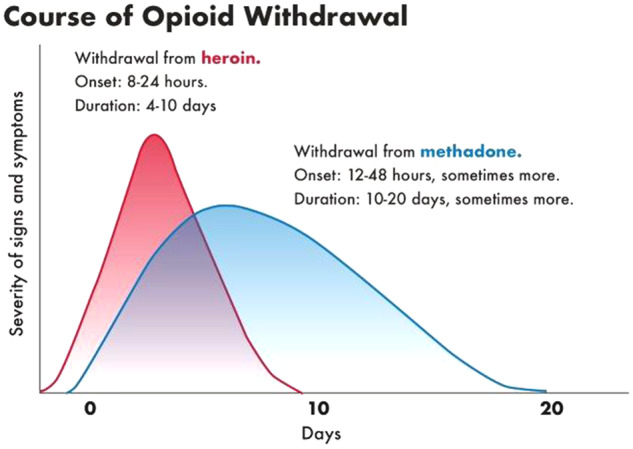
**Course of opioid withdrawal.** The figure shows different time course for opioids heroine (red curve) and methadone (blue curve). Although heroin half-life is 2–6 min, the withdrawal symptoms start usually at 6–24 h after the last dose, reach a peak at 24–48 h, and resolve after 4–10 days, although this delay as already mentioned, maybe explained by the fact that the main active molecule in the brain after heroin administration is not heroin itself but rather morphine and 6-acetylmorphine, which have much longer half-lives and thus longer-lasting intoxication and withdrawal; for methadone, with a half-life of 15–55 h, the withdrawal symptoms start usually at 36–48 h after the last dose, the withdrawal may be prolonged, and last 3–6 weeks. Adapted from Drug and Alcohol Withdrawal Clinical Practice Guidelines—NSW (New South Wales Government, 2008).

**Figure 2:**
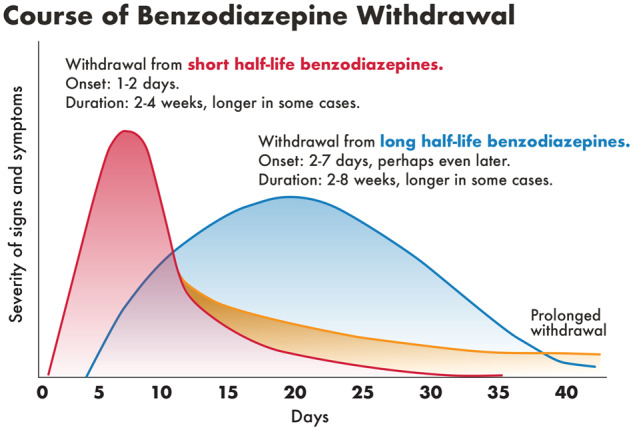
**Course of BZ withdrawal.** The figure shows different time course for short (red curve) and long half-life BZ (blue curve) which is related to the drugs half-lives. It shows also a formation of protracted withdrawal (yellow curve) for short half-life BZ. Adapted from Drug and Alcohol Withdrawal Clinical Practice Guidelines—NSW (New South Wales Government, 2008).

Some investigators consider that withdrawal symptoms last only for a maximum of five half-lives of the drug, which is obviously incorrect, although it is not clear where this statement originated as we did not find the source. For example, heroin half-life is 2-6 min, but duration of the withdrawal syndrome is approximately 4–10 days (see Fig. 1), although this delay may be explained by the fact that the main active molecule in the brain after heroin administration is not heroin itself but rather morphine and 6-acetylmorphine, which have longer half-lives and thus intoxication and withdrawal is longer lasting (Inturrisi *et al.*, 1983; Rook *et al.*, 2006). For amphetamines, half-life is ∼9–11 h, whereas withdrawal symptoms only start 2–4 days after drug discontinuation (New South Wales Government, 2008) and last at least 2–4 weeks. Generally, acute withdrawal symptoms for drugs with very short lifetimes (e.g. heroin) last about a week; for drugs with a medium half-life of ∼10–20 h (e.g. short-acting BZ), the acute withdrawal syndrome may last 2–4 weeks; whereas for drugs with longer half-lives (e.g. long-half-life BZ) the withdrawal phase may last 2–8 weeks (Fig. 2). However, this generalization applies only to acute withdrawal syndrome; symptoms of protracted withdrawal may last for weeks and months.

### Current theories on the neurobiological basis of dependence and withdrawal

#### Opponent process theory of drug withdrawal

Because few drug groups discussed in this manuscript namely opioids, stimulants and BZ are also involved in addictive disorders alternative concepts of dependence and withdrawal are presented (Koob and Le Moal, 2008*a*, *b*).

Addiction is depicted as a compulsive disorder with excessive drug intake and loss of control over intake, with decreases in reward neurotransmission and activation of brain stress systems. Addiction is a disorder that involves elements of both impulsivity and compulsivity in which impulsivity predominates at the early stages of addiction (preoccupation and anticipation) whereas compulsivity dominates at the final stages of withdrawal and negative affect.

The key brain structures involved in these processes are ventral striatum with neurotransmitters dopamine and opioid peptides and extended amygdala with corticotropin-releasing factor, norepinephrine and dynorphin. In fact, it is proposed that all main drugs of abuse act through the central nucleus of the amygdala producing increases in reward thresholds, anxiety-like responses and extracellular levels of corticotropin-releasing factor. Specifically, during development of dependence and withdrawal brain antireward systems are recruited and include corticotropin-releasing factor, norepinephrine and dynorphin which produce aversive states such as dysphoria and stress. The components of acute drug withdrawal include decrease of activity of the mesocorticolimbic dopaminergic system and decreased activity in the nucleus accumbens/amygdala mediated by opioid peptides, GABA and glutamate.

### Summary points

- Dependence and withdrawal are results of neuroadaptation that occur during drug treatment.
- Dependence and withdrawal are not equated with drug addiction although may coexist.
- The shortest known time to acquire dependence occurs with opioid drugs where 2–3 weeks or longer exposure at the therapeutic dose may produce dependence and upon drug discontinuation or exposure to antagonist (naloxone) also an acute withdrawal syndrome.

## Part II: Specific acute withdrawal syndromes acute withdrawal syndrome—general features

Ries *et al.* (2014) report the American Society of Addiction Medicine (ASAM) definition of withdrawal as ‘The onset of a predictable constellation of signs and symptoms following the abrupt discontinuation of, or rapid reduction in, the dose of a psychoactive substance’.

Chouinard and Chouinard (2015) describe in a very comprehensive manner general features of the emergence of new, acute withdrawal symptoms after drug discontinuation.

1. New withdrawal symptoms include the symptoms that first appeared during the drug discontinuation or dose decrease and are not related to symptoms of the patient’s original illness.
2. Different classes of CNS drugs have some common withdrawal symptoms, as well as some specific to the drug class withdrawal symptoms.
3. New withdrawal symptoms which are common to many CNS drugs include: nausea, anxiety, headaches, decreased concentration, irritability, agitation, tremor, sleep disturbances, dysphoria and depression.
4. Some new withdrawal symptoms may be characteristic of a specific CNS drug class:
  • Lacrimation, rhinorrhoea and sneezing for opioids
  • Electric-shock sensations, confusion and myoclonus for SSRIs.
5. The new withdrawal symptoms are usually transient and reversible.
6. Major complications of abrupt withdrawal may occur, such as seizures and psychoses in cases of BZ withdrawal (Preskorn and Denner, 1977; Petursson, 1994), seizures in cases of sertraline (Zoloft label) and zolpidem withdrawal (Aragona, 2000), and suicidality involving antidepressants (Yerevanian *et al.*, 2004; Valuck *et al.*, 2009) and MAO inhibitors such as phenelzine (Dilsaver, 1994).
7. The onset of withdrawal symptoms depends on the duration of action; usually, short-acting drugs have an early peak of onset.
8. Drugs with higher potency and short/intermediate half-life have greater risks for formation of drug dependence and withdrawal symptoms.

There are well-known withdrawal syndromes in different classes of scheduled and unscheduled drugs. These are described in detail below, along with symptoms and time course of withdrawal syndrome.

Withdrawal syndromes for scheduled drug classes apply to:

- Opioids
- Benzodiazepines
- Z-Drugs (Zolpidem, Zaleplon and Eszopiclone)
- Barbiturates
- Stimulants (amphetamine, cocaine and methamphetamine)
- Testosterone and androgenic anabolic steroids
- Ketamine
- Cannabis

Withdrawal syndromes for unscheduled drug classes apply to:

- Antidepressants: SSRIs and serotonin-norepinephrine reuptake inhibitors (SNRIs)
- Tricyclic antidepressants
- Antipsychotics
- Anti-Parkinsonian drugs
- Monoamine oxidase inhibitors
- Gabapentin
- Nicotine/tobacco
- Corticosteroids
- Statins
- Aspirin

### Acute withdrawal syndromes—scheduled drugs—examples

#### Opioid withdrawal syndrome

The first descriptions of opioid withdrawal are those by Light and Torrence (1929) and Himmelsbach (1939). The symptoms include yawning, rhinorrhoea, piloerection, perspiration, lacrimation, mydriasis, hand tremors, hot and cold flashes, restlessness, muscle twitches, abdominal cramps, anxiety and chills, myalgia, irritability, backache, joint pain, weakness, insomnia, nausea, anorexia, vomiting, diarrhoea, or increased blood pressure, respiratory rate or heart rate (Light and Torrence, 1929; Himmelsbach, 1939; Handelsman *et al.*, 1987), morphine label (morphine sulphate extended-release tablets, morphine sulfate Contin, label 2018; Table 1). Symptoms of withdrawal may start as early as 6–24 h after the last dose (heroin), peak at 24–48 h, and resolve after 5–10 days.

**Table 1:** Summary table for the most common withdrawal syndromes

Adverse events	Opioids	BZ	Stimulants	SSRIs-SNRIs	Cannabis	Nicotine	Anti-psychotics	Androgenic anabolic steroids and testosterone
Acute withdrawal syndrome								
Anxiety	+	+	+	+	+	+	+	+
Depression/depressed mood		+	+	+	+	+		+
Irritability	+	+	+		+	+		+
Agitation		+	+	+			+	
Insomnia	+	+	+	+	+	+	+	+
Somnolence/lethargy			+	+		+		+
Psychotic behaviours		+						+
Hallucinations		+	+	+				
Suicidality (rare)		+	+	+				+
Tremors	+	+			+		+	
Restlessness	+	+	+		+	+	+	+
Seizures	+ Neonates	+						
Nausea/vomiting	+	+		+			+	
Diarrhoea	+			+			+	
Constipation						+		
Appetite		↓	↑	↓	↓	↑	↓	↓
Weight		↓			↓	↑		
Heart rate	↑	↑	↓	↑		↓		
Blood pressure	↑	↓Postural						
Sweating	↑	↑			↑	↑	↑	
Drug group specific AEs	Piloerection/mydriasis/yawning/rhinorrhea	Depersonalization/derealization/delirium	Vivid, unpleasant dreams/paranoid ideation	Brain zaps/ derealization/depersonalization/myoclonus	Disturbing dreams/abdominal pain		Rhinorrhea/rare NMS	Decreased libido/hypogonadotropic hypogonadism
**Acute withdrawal syndrome.** Onset Duration	+ 6 h to 2 days 10 days to 6 weeks	+ 2–7 days 2–8 weeks	+ 1–4 days 1–4 weeks	+ 36–96 h ≤6 weeks	+ 1–4 days 2–3 weeks	+ 24–72 h 2–4 weeks	+ 36–96 h ≤6 weeks	+ INF
**Rebound** Peak onset Duration		+ 1–5 days^a^ Insomnia ≥3 nights Anxiety ≥ 2 weeks	+ hours-attention-deficit/hyperactivity disorder INF	+ 36–96 h ≤6 weeks			+ 36–96 h ≤6 weeks	
**Protracted withdrawal** Onset after drug discontinuation Duration	+ ∼6–9 weeks 6–9 months	+ ∼4–6 weeks 6–12 months	+ ∼2–4 weeks Weeks to months	+ ∼24 h to 6 weeks Months or longer			+ ∼24 h to 6 weeks Months or longer	+ weeks to months

a Rebound insomnia is specific to short and intermediate half-life BZ compounds.

+ , symptom/syndrome present; ↑, symptom increased; ↓, symptom decreased; BZ = benzodiazepines; INF = information not found; NMS = neuroleptic malignant syndrome; SSRIs = selective serotonin reuptake inhibitors; SNRIs = selective serotonin-norepinephrine reuptake inhibitors.

For long-acting opioid (methadone) withdrawal starts after 36–48 h after the last dose, and may last 3–6 weeks (New South Wales Government, 2008).

The withdrawal symptoms of oxycodone products include restlessness, lacrimation, rhinorrhoea, yawning, perspiration, chills, myalgia and mydriasis, also other symptoms may develop such as irritability, anxiety, backache, joint pain, weakness, abdominal cramps, insomnia, nausea, anorexia, vomiting, diarrhoea, increased blood pressure, respiratory rate and heart rate (Oxycontin label Sep 2018, Roxybond label April 2017).

Opioid withdrawal symptoms have been also observed in children undergoing treatment with opioids. In these cases, physical dependence developed as early as 2–3 days following continuous opioid therapy; the most commonly seen symptoms of withdrawal in children included:

1. Neurological adverse events such as anxiety, agitation, grimacing, insomnia, increased muscle tone, abnormal tremors and choreoathetoid movements.
2. Gastrointestinal symptoms included vomiting, diarrhoea and poor appetite.
3. Autonomic signs included tachypnoea, tachycardia, fever, sweating and hypertension (Ista *et al.*, 2008; Anand *et al.*, 2010; Fisher *et al.*, 2013).

Neonatal abstinence syndrome (NAS) is unusual in that the withdrawal symptoms are related to the maternal opioid intake but then manifest in the newborn. The clinical presentation of NAS includes signs of neurological excitability such as tremors, hyperirritability, increased wakefulness, excessive high-pitched crying, increased muscle tone, hyperactive deep tendon reflexes, exaggerated Moro reflex, seizures, frequent yawning and sneezing, and also symptoms of gastrointestinal dysfunction such as poor feeding, unco-ordinated and constant sucking, hyperphagia, vomiting, diarrhoea and dehydration. Onset varies with the opioid pharmacology, in case of maternal use of heroin NAS may start 24 h of birth, whereas in case of withdrawal from methadone NAS usually starts around 24–72 h of age (Hudak *et al.*, 2012; Kocherlakota, 2014).

Although generally not life-threating, in some cases opioid withdrawal may lead to serious adverse events such as Takotsubo cardiomyopathy ‘broken heart syndrome’ in cases of methadone and extended-release long-acting oxycodone withdrawal (Rivera *et al.*, 2006; Lemesle *et al.*, 2010; Saiful *et al.*, 2011; Spadotto *et al.*, 2013), or seizures due to neonatal abstinence syndrome (Hudak *et al.*, 2012; Kocherlakota, 2014).

The time course of the severity of acute withdrawal symptoms for opioid drugs depends usually on the half-life of the drug, although it can be quite variable. Figure 1 shows onset, peak, duration and severity of acute withdrawal symptoms for two frequently used and abused opioids, heroin and methadone (New South Wales Government, 2008).

It is important to note that opioid withdrawal also includes a protracted phase of withdrawal which occurs after the acute withdrawal phase and is further discussed in the ‘Protracted Withdrawal Syndrome’ section.

As mentioned in the ‘Definitions of dependence and withdrawal’ section, there is a difference between withdrawal syndrome and addiction.

Opioid addiction is a complex disorder and its development can vary. An individual’s risk in developing addiction likely relates to a combination of health, social, economic, lifestyle and genetic factors. A history of substance abuse, psychiatric disorders, mistreatment, poverty, and access to prescription or illegal opioids may also contribute to a person’s risk of opioid addiction (Webster *et al.*, 2011).

### Benzodiazepine withdrawal syndrome

BZ withdrawal symptoms may vary in intensity. Mild withdrawal may include anxiety, apprehension, fearfulness, insomnia, irritability, agitation, restlessness, dizziness, headache, anorexia, weight loss, difficulty in concentration, muscle stiffness and pain, hyperosmia, metallic taste, perceptual changes, sweating and intolerance to light and sound. More severe symptoms include nausea, vomiting, vertigo, cramps, weakness, tremor, tachycardia, postural hypotension, hyperthermia, panic attacks, depression, psychotic reactions, depersonalization, de-realization, delirium, delusions and hallucinations (MacKinnon and Parker, 1982; Petursson, 1994; New South Wales Government, 2008). The most serious adverse events which may occur are psychosis, delirium, suicidality, seizures and in older people catatonia (Preskorn and Denner, 1977; Owen and Tyrer, 1983; Risse *et al.*, 1990; Lader, 1994; Petursson, 1994; Rosebush and Mazurek, 1996; Lebin and Cerimele, 2017; Reeves and Kamal, 2019; Table 1).

The timing of withdrawal symptoms depends on several factors; the main one is BZ half-life. Long-acting BZs include diazepam, chlordiazepoxide, flurazepam and clorazepate; shorter-acting BZs include oxazepam, lorazepam and triazolam. For the long-acting BZs, there is a lag period of 3–7 days for onset of withdrawal symptoms following the drug discontinuation; whereas the short-acting BZs symptoms of withdrawal may occur within 24 h. The correlation between drug half-life and onset of symptoms and length of the withdrawal is presented in Fig. 2, below (New South Wales Government, 2008). Figure 2 also shows formation of protracted withdrawal which starts at the end of the acute withdrawal phase.

Use of BZ during the later stages of pregnancy can result in withdrawal symptoms in the neonate such as apnoea, bradycardia, hypertonia, irritability, hypothermia, hyperactivity, tachypnoea, restlessness, tremors, hyperreflexia, inconsolable crying, cyanosis, diarrhoea, vomiting and feeding difficulties (Iqbal *et al.*, 2002; Hudak *et al.*, 2012).

### Z-drugs withdrawal syndrome (zolpidem and zopiclone)

Dependence following the use of zolpidem has been known since the 1990s as occurring mainly in people abusing it. There are also a considerable number of zolpidem dependence case reports in the scientific literature (Aragona, 2000; Tripodianakis *et al.*, 2003; Cubala and Landowski, 2007; Victorri-Vigneau *et al.*, 2007; Victorri-Vigneau *et al.*, 2014; Chattopadhyay *et al.*, 2016).

The withdrawal syndrome after abrupt withdrawal of zolpidem was already noted during the clinical studies of Ambien (zolpidem tartrate). Adverse events reported within 48 h of drug discontinuation included fatigue, nausea, flushing, lightheadedness, uncontrolled crying, emesis, stomach cramps, panic attack, nervousness, abdominal discomfort, hyperventilation, shallow breathing, angry feelings, cramping muscles and restlessness (Watsky, 1996; Sethi and Khandelwal, 2005) and labels for zolpidem (e.g. Ambien, Edluar 2019). Withdrawal seizures after zolpidem discontinuation were reported mainly in cases of use of supratherapeutic doses (Aragona, 2000; Tripodianakis *et al.*, 2003; Sethi and Khandelwal, 2005; Cubala and Landowski, 2007).

Also, withdrawal symptoms after discontinuation of zopiclone were reported, and included cravings, rebound insomnia, anxiety or panic attacks, weakness, palpitations, tachycardia, tremor and withdrawal seizures. These symptoms were mainly seen in subjects using supratherapeutic doses; rebound insomnia and rebound anxiety were also noted (Cimolai, 2007).

### Barbiturate withdrawal syndrome

Abrupt discontinuation of a barbiturate after prolonged use or abuse can result in a withdrawal syndrome which is sometimes life-threatening. Withdrawal symptoms include weakness, tremor, muscle twitches, agitation, anxiety, anorexia, nausea, vomiting, weight loss, disorientation in time and place, agitation, tremulousness, insomnia, delusions, visual and auditory hallucinations, postural hypotension and high fever. The most serious adverse events of withdrawal are delirium, generalized convulsions and cardiovascular collapse that may lead to death; however, severe withdrawal occurs mainly after dependence on short- or intermediate-acting barbiturates such as pentobarbital, secobarbital, amobarbital or butalbital (Fraser *et al.*, 1953; Essig, 1968; Gault, 1976; Gussow and Carlson, 2013).

Also, neonatal withdrawal syndrome associated with barbiturates has been identified.

Infants born to mothers physically dependent on barbiturates may develop dependence and subsequently experience withdrawal. The withdrawal may manifest within the first few days of life and symptoms include excessive crying, hyperphagia, vomiting, diarrhoea, tremors, irritability, hyperacusis, vasomotor instability, sweating, restlessness, sleeplessness, increased tone and sometimes seizures (Desmond *et al.*, 1972; Hudak *et al.*, 2012; Gresham and LoVecchio, 2015) and label Butisol (2019).

### Stimulant withdrawal syndrome

**Figure 3:**
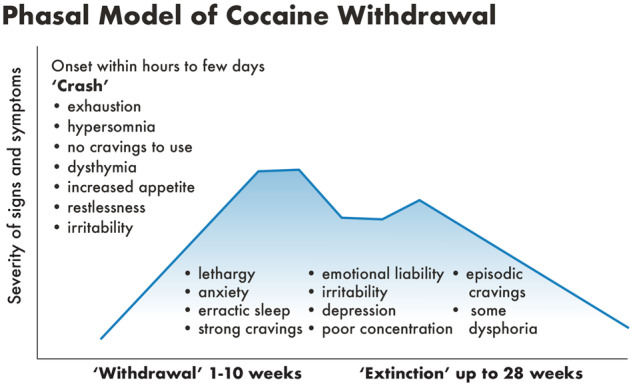
**Phasal model of cocaine withdrawal.** The figure shows three phases of cocaine withdrawal: crash, withdrawal and extinction, which have somewhat different symptoms profile. Adapted from Baker *et al.* (2004).

Withdrawal from stimulants such as amphetamine (including dexamphetamine and methamphetamine), MDMA (ecstasy) and cocaine usually occurs in three phases (crash, withdrawal and extinction phase), Fig. 3. The crash phase, starts as stimulants wear off, can last for several days and includes fatigue, flat affect, increased sleep and reduced cravings; severe depressive symptoms may occur. The withdrawal phase typically starts 2–4 days after the last amphetamine use and 1–2 days after the last cocaine use. Withdrawal phase symptoms may include strong cravings, fluctuating mood and energy levels, psychomotor retardation or agitation, irritability, restlessness, anxiety, fatigue, lack of energy, anhedonia, vivid and unpleasant dreams, insomnia or hypersomnia, general aches and pains, headaches, muscle tension, increased appetite, poor concentration and attention, disturbances of thought (paranoid ideation, strange beliefs) and hallucinations; bradycardia may be present and is a reliable measure of stimulant withdrawal (Kosten and O’Connor, 2003; New South Wales Government, 2008; APA, 2013; Table 1). The withdrawal symptoms gradually decrease over 2–4 weeks for amphetamine and 1–2 weeks for cocaine, although some symptoms persist during the extinction phase/protracted withdrawal.

Withdrawal from stimulant drugs is generally not medically dangerous; however, depressive symptoms with suicidal ideation or behaviour may occur and are generally the most serious problems seen during the stimulant withdrawal (APA, 2013).

### Ketamine withdrawal syndrome

Ketamine is known for the formation of severe psychological dependence and significant, rapidly developing tolerance (Kamaya and Krishna, 1987; Hurt and Ritchie, 1994; Jansen and Darracot-Cankovic, 2001; Pal *et al.*, 2002) without a prominent physical withdrawal syndrome. However, the presence of physical withdrawal symptoms after ketamine discontinuation has also been described, and includes craving, dysphoria, shaking, sweating, palpitations, tiredness, low appetite, low mood, chills, autonomic arousal, lacrimation, restlessness, anxiety, nightmares, paranoia, delusions and hallucinations (Lim, 2003; Critchlow, 2006; Chen *et al.*, 2014). Addiction websites (American Addiction Centers and Addiction Center) additionally list the following adverse events occurring in ketamine abusing subjects during the withdrawal period: agitation, confusion, psychosis, including delusion and hallucination; loss of motor skills, rage, nausea, decreased respiratory and cardiac functions, insomnia, cognitive impairment, loss of appetite, tremors, restlessness, depression, nightmares and chills.

Timelines of ketamine withdrawal include: (i) acute withdrawal symptoms that typically begin within 24 h of discontinuing ketamine use and last approximately 3 days; symptoms include shaking, fatigue, insomnia, rage, depression, hallucinations, delusions, tremors, nausea and rapid breathing; (ii) withdrawal symptoms that may persist for 2 weeks, but begin to decrease; and (iii) after approximately 2 weeks, most withdrawal symptoms usually stabilize.

### Testosterone and anabolic-androgenic steroids withdrawal syndrome

The withdrawal syndrome after a chronic intake of supratherapeutic doses of testosterone and androgenic anabolic steroids can last for weeks or months and is characterized by depressed mood, major depression, suicidality, fatigue, craving, headache, muscle and joint pain, anorexia, anxiety, restlessness, irritability, insomnia or hypersomnia, decreased libido, hypogonadotropic hypogonadism and suppression of the hypothalamic–pituitary–testicular (HPT) axis (Thiblin *et al.*, 1999; Medras and Tworowska, 2001; Brower, 2002, 2009; Kanayama *et al.*, 2009) and Testosterone label, 2017 (Table 1).

### Cannabis withdrawal syndrome

Withdrawal symptoms of cannabis include irritability, anger, aggression, decreased appetite, weight loss, nervousness, anxiety, restlessness, sleep difficulties including insomnia and strange or disturbing dreams, abdominal pain, shakiness, tremors, fever and headache. Less common symptoms include chills, depressed mood and sweating (Budney *et al.*, 2004; Budney and Hughes, 2006; New South Wales Government, 2008; Gorelick *et al.*, 2012; APA, 2013; Bonnet and Preuss, 2017; Schlienz *et al.*, 2017; Table 1).

**Figure 4:**
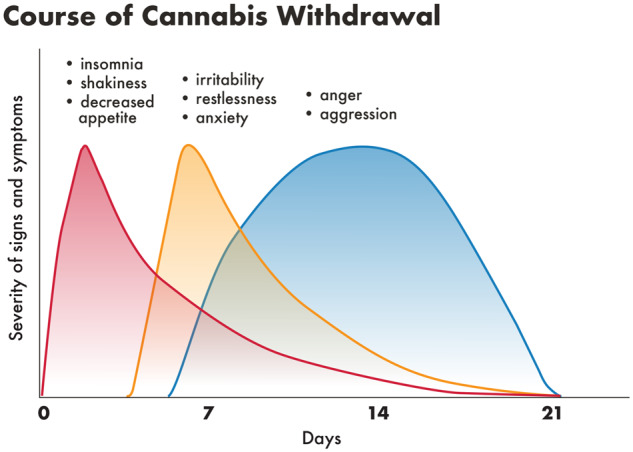
**Course of cannabis withdrawal.** The figure shows different phases of occurrence of cannabis withdrawal symptoms, with the early phase (red curve) of insomnia, shakiness, decreased appetite symptoms, middle phase (yellow curve) with irritability, anxiety, restlessness symptoms and late somewhat prolonged phase (blue curve) of anger and aggression. Adapted from Drug and Alcohol Withdrawal Clinical Practice Guidelines – NSW (New South Wales Government, 2008).

Withdrawal symptoms usually start within the first 1–4 days of abstinence, peak within the first week, and then resolve within 2–3 weeks of drug discontinuation (Schlienz *et al.*, 2017). Strange dreams and sleep difficulties can persist longer (Budney and Hughes, 2006; Herrmann *et al.*, 2015). Also, there is temporal variation in the occurrence of specific symptoms, with the early onset of insomnia and shakiness, followed by irritability and anxiety (day 4–5) and anger and aggression peaking after 2 weeks of abstinence (Fig. 4) setting. The timing of the severity of withdrawal symptoms to some degree reflects the changes at the cannabinoid 1 (CB1) receptors level in the brain where regular cannabis intake causes a desensitization and down-regulation of CB1 receptors, and during the first 2 days of withdrawal this begins to reverse and the receptors begin to return to normal functioning within 4 weeks of abstinence (Bonnet and Preuss, 2017).

### Acute withdrawal syndromes—non-scheduled drugs—examples

#### Antidepressant SSRIs and SNRIs withdrawal syndrome

Some more frequently used SSRIs include: fluoxetine, fluvoxamine, paroxetine, citalopram and sertraline. Some more frequently used SNRIs include: venlafaxine, desvenlafaxine and duloxetine.

The SSRIs and SNRIs withdrawal syndrome is characterized by the following symptoms, general: headaches, flu-like symptoms, tachycardia; gastrointestinal: nausea, vomiting, diarrhoea, anorexia; sleep-related: vivid dreams, nightmares, insomnia or hypersomnia; neuropsychiatric: anxiety, depression, suicidality, hypomania, agitation, dysphoria, aggression, hallucinations, de-realization and depersonalization, decreased concentration, dizziness, ataxia, electric-shock sensations (‘brain-zaps’), confusion and myoclonus (Leiter *et al.*, 1995; Rosenbaum *et al.*, 1998; Haddad, 2001; Yerevanian *et al.*, 2004; Chouinard and Chouinard, 2015; Fava *et al.*, 2015) and labels e.g. Paxil, 2014; Prozac, 2017; Zoloft, 2017, Table 1. SSRIs and SNRIs withdrawal symptoms have their peak of onset at 36–96 h or later, depending on the drug’s half-life, and may last up to 6 weeks, also depending on drug half-life (Chouinard and Chouinard, 2015). The individual SSRIs have somewhat different profiles of adverse events, and it seems that severity might be related to half-life (Rosenbaum *et al.*, 1998; Fava *et al.*, 2015). Also, it was observed that withdrawal syndrome was more common in young patients than in the elderly (Himei and Okamura, 2006; Fava *et al.*, 2015). Frequently, acute withdrawal syndrome may be followed by disease rebound (see Rebound section) and protracted withdrawal syndrome (see Protracted Withdrawal Syndrome section).

Of note, there is a tendency in the scientific literature to call SSRIs and SNRIs withdrawal syndrome a ‘discontinuation syndrome’, which in our opinion is both scientifically incorrect and misleading, as it may suggest an absence of the withdrawal syndrome. A comment by Chouinard and Chouinard (2015) explains this issue well: ‘The terminology discontinuation refers to the medical prescribing act or a patient’s self-discontinuation of medication. Furthermore, the term discontinuation syndrome is misleading since withdrawal may occur without discontinuation, for example, in between two doses of rapid-onset and short-acting drugs (e.g. clock watching syndrome) and with a decrease in medication’. Fava *et al.* (2015) adds, ‘The term “discontinuation syndrome” minimizes the vulnerabilities induced by SSRI and should be replaced by “withdrawal syndrome”’. However, discussion on the topic of this nomenclature continues in the scientific literature (Schatzberg *et al.*, 1997; Shelton, 2006; Nielsen *et al.*, 2012; Chouinard and Chouinard, 2015; Fava *et al.*, 2015).

In addition, neonatal withdrawal syndrome associated with SSRIs has been identified. It includes respiratory distress, cyanosis, seizures, feeding difficulty, vomiting, hypoglycaemia, hypotonia or hypertonia, hyperreflexia, tremor, jitteriness, irritability, pulmonary hypertension; and with paroxetine use, necrotizing enterocolitis (Stiskal *et al.*, 2001; Sanz *et al.*, 2005; Koren and Boucher, 2009; Hudak *et al.*, 2012) and SSRI labels e.g. Paxil, 2014; Prozac, 2017; Zoloft, 2017.

#### Tricyclic antidepressant withdrawal syndrome

Some more frequently used tricyclic antidepressants include: imipramine, clomipramine, amitriptyline, amoxapine, desipramine, doxepin and nortriptyline. Tricyclic antidepressant withdrawal symptoms can be grouped into four discrete syndromes: (i) gastrointestinal and general somatic distress with occasional anxiety and agitation, also nausea, vomiting, diarrhoea, abdominal pain and anorexia; (ii) sleep disturbances, such as initial and middle insomnia, vivid dreams and nightmares; (iii) parkinsonism including bradykinesia, cogwheel rigidity, tremor or akathisia; and (iv) paradoxical mania. Sometimes withdrawal syndrome presents as a ‘flu-like’ syndrome; which in addition to gastrointestinal symptoms, also includes diaphoresis, dizziness, chills, malaise, fatigue, myalgia and headache; also suicidality and delirium may occur (Dilsaver, 1990; Ceccherini-Nelli *et al.*, 1993; Garner *et al.*, 1993; Dilsaver, 1994; Haddad, 2001; Yerevanian *et al.*, 2004) and label Anafranil, 2014.

In paediatric populations, the most frequent symptoms are irritability, decreased appetite, drowsiness, fatigue, insomnia; also nausea, vomiting and abdominal cramps, which, if severe, may lead to dehydration (Garner *et al.*, 1993).

There are also neonatal withdrawal symptoms that are attributed to maternal use of tricyclic antidepressants; these adverse events include irritability, respiratory difficulty, hypothermia, poor feeding, tremors, jitteriness, myoclonus and hyperactive Moro reflex. Serious withdrawal reactions may be present, such as tachycardia, cyanosis and convulsions (Cowe *et al.*, 1982; Bloem *et al.*, 1999; Haddad, 2001; Hudak *et al.*, 2012; ter Horst *et al.*, 2012) and label Anafranil (2014).

#### Antipsychotic drug withdrawal syndrome

Antipsychotic drugs belong to multiple pharmacological groups: phenothiazines, thioxanthenes, butyrophenones and atypical antipsychotics. For these drugs, the common withdrawal symptoms are nausea, vomiting, diarrhoea, anorexia, influenza-like syndrome, rhinorrhoea, diaphoresis, myalgia, paraesthesia, anxiety, agitation, restlessness, insomnia, vertigo and tremor (Dilsaver, 1994; Borison, 1996; Shiovitz *et al.*, 1996) and labels e.g. Clozaril February 2017, Table 1. Symptoms may appear 36–96 h or later after drug discontinuation, dose reduction or switch, depending on the drug’s duration of action and last up to 6 weeks (Chouinard *et al.*, 2017). Of note is that during the withdrawal period, not uncommon is also psychosis rebound which results in re-emergence or worsening of psychosis (see Rebound section) or supersensitivity psychosis and occurrence of withdrawal-emergent tardive dyskinesia (Crane, 1968; Chouinard and Jones, 1980; Borison *et al.*, 1988; Schultz *et al.*, 1995; Margolese *et al.*, 2005; Karas *et al.*, 2016; Chouinard *et al.*, 2017).

A rare, but potentially life-threatening reaction with 10% mortality rate (Strawn *et al.*, 2007), to antipsychotic withdrawal is a neuroleptic malignant syndrome (NMS), characterized by hyperthermia, muscular rigidity, rhabdomyolysis, autonomic instability, mental status changes, diaphoresis, incontinence and elevated creatine phosphokinase (Corrigan and Coulter, 1988; Kasckow *et al.*, 1990; Spivak *et al.*, 1990; Amore and Zazzeri, 1995).

Anti-psychotics taken during the third trimester of pregnancy may cause neonatal withdrawal symptoms which may include agitation, tremor, somnolence, hypertonia, hypotonia, respiratory distress and feeding disorder (e.g. label Risperdal 2019; Haldol 2019, Clozaril 2017).

#### Levodopa-withdrawal malignant syndrome (withdrawal-emergent hyperpyrexia and confusion)

This is an uncommon, but potentially fatal complication (Sechi *et al.*, 1984) which follows reduction or cessation of anti-parkinsonian medications, including levodopa, dopamine agonists, and even amantadine (Friedman *et al.*, 1985; Rosse and Ciolino, 1985; Rainer *et al.*, 1991; Mizuno *et al.*, 2003; Serrano-Duenas, 2003; Brantley *et al.*, 2009; Newman *et al.*, 2009) and labels e.g. Apokyn 2004, Parlodel 2012, Sinemet 2017, Requip 2017. Typically, symptoms develop between 18 h and 7 days following anti-parkinsonian drug discontinuation. Clinical features include high fever, marked rigidity, consciousness disturbances ranging from drowsiness to coma, autonomic dysfunction such as tachycardia, perspiration, non-paralytic ileus, blood pressure fluctuation, rhabdomyolysis and elevation of serum creatinine phosphokinase. Symptoms are similar to those of NMS.

Amantadine withdrawal may precipitate NMS also in patients with other than Parkinson’s disease, namely with influenza A encephalopathy (Ito *et al.*, 2001).

#### Dopamine agonist withdrawal syndrome in Parkinson’s patients

The syndrome was described by Rabinak and Niremberg (2010) in Parkinson’s disease patients who were withdrawn from long-term treatment with dopamine agonists due to the development of impulse control disorders. The syndrome presents as a constellation of neuropsychiatric and autonomic symptoms: depression, anxiety, agoraphobia, fatigue, dysphoria, irritability, agitation, pain, sleep disturbances, diaphoresis, orthostatic hypotension and drug cravings.

#### Nicotine/tobacco withdrawal syndrome

The nicotine withdrawal syndrome includes subsets of somatic components, cognitive, and affective components; the somatic signs include bradycardia, constipation, increased appetite and weight gain; affective symptoms include depressed mood and anhedonia, anxiety, irritability, frustration, dysphoria, emotional lability, anger, other symptoms are restlessness, insomnia, fatigue, somnolence, difficulty concentrating, sweating, confusion and craving. Tobacco/nicotine withdrawal usually begins within 24–72 h of stopping or decreasing tobacco use, peaks at 2–3 days after abstinence, and lasts 2–4 weeks (Hughes, 2007; New South Wales Government, 2008; APA, 2013; Jackson *et al.*, 2015) and label Nicotrol 2010, Table 1.

#### Treatment and prevention of acute withdrawal syndrome

For the drugs with known acute withdrawal syndromes, the main treatments and prevention measures are: (i) taper at the end of treatment; (ii) after abrupt drug discontinuation immediate substitute with the drug with similar mechanism of action or reinstitute the discontinued drug, if possible, and taper much slower; (iii) treat symptomatically the dangerous or unpleasant symptoms such as seizures, psychosis, depression, headaches, vomiting, diarrhoea (Ashton, 1994; Kosten and O’Connor, 2003; O'Brien C, 2005; New South Wales Government, 2008; Chouinard and Chouinard, 2015; Chouinard *et al.*, 2017).

The above presented list of acute withdrawal syndromes is by no means complete; it only represents better known syndromes. However, it is important to emphasize that the list of new withdrawal syndromes is growing along with new drug approvals and the identification of new withdrawal syndromes for older drugs that are frequently recognized only during the post-marketing period.

The reason for listing the above non-scheduled drugs, which are used by innumerable numbers of patients, is to point out that these drugs not only can produce dependence, withdrawal and rebound; but in some cases, these adverse events may be life-threatening, and the labels do not necessarily reflect this fact and provide necessary warnings for patients and physicians.

We provide the additional listing of acute withdrawal syndromes in the Supplementary material 2 for less frequently used drugs and/or few not CNS-active drugs although frequently used including monoamine oxidase inhibitors, gabapentin, corticosteroids, statins and aspirin.

In Table 1 are listed the most frequent and relevant acute withdrawal syndromes, rebound and protracted withdrawal syndromes.

### Summary points

- Majority of CNS-active drugs, scheduled and not scheduled, produce acute withdrawal syndromes.
- Onset and duration of withdrawal depends on drugs’ duration of action and half-life.
- The characteristic of withdrawal syndrome mainly depends on neurotransmitter system affected, and generally presents opposite symptoms to that seen during drug administration.

## Part III. Rebound phenomena—definition and examples

Another aspect of withdrawal, rebound effect, also called rebound phenomenon, occurs in a similar timeframe to that of acute withdrawal. Rebound phenomenon is a rapid return of the patient’s original symptoms at a greater intensity than before the treatment. Rebound may cause in a small subset of susceptible individuals serious and fatal adverse events after abrupt drug discontinuation, such as severe psychosis after neuroleptics (Chouinard *et al.*, 2017), severe worsening of multiple sclerosis after immunomodulatory drugs (Gonzalez-Suarez *et al.*, 2017; Larochelle *et al.*, 2017) or suicidality after antidepressants (Valuck *et al.*, 2009). However, unlike protracted withdrawal syndromes rebound symptoms are transient and reversible and return after days or weeks to the baseline.

Lupolover *et al.* (1982) reviewed several CNS-active and non-CNS-active drugs and provided the following conclusion. ‘The survey of the medical literature with regard to the “rebound phenomenon” after withdrawal of drugs shows that it can appear after all classes of drugs, irrespective of their chemical formulation or pharmacological action. Cardiovascular drugs, diuretics, hormones, anticoagulants, antacids and neuropsychiatric drugs have been said to produce “rebound phenomena” on withdrawal’.

Emergence of rebound is variable for different drugs and may be seen in only 36–96 h for SSRIs and oral antipsychotics (Chouinard and Chouinard, 2015; Chouinard *et al.*, 2017) and in 1–5 days for some BZ such as in patients with anxiety experiencing rebound after abrupt discontinuation of bromazepam and diazepam (Fontaine *et al.*, 1984) and Fig. 6 and in the cases of patients with insomnia after abrupt withdrawal of triazolam (Kales *et al.*, 1987) or lorazepam (Scharf *et al.*, 1982).

**Figure 5:**
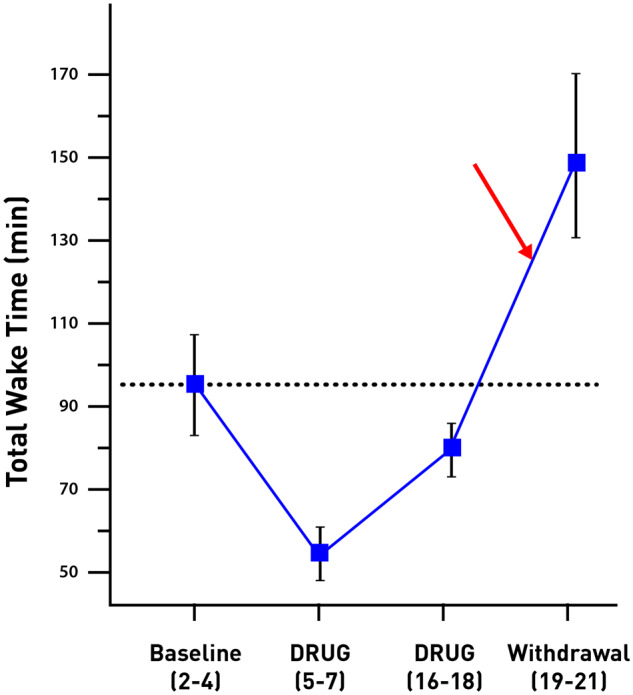
**Rebound insomnia in patients treated with triazolam after abrupt drug withdrawal.** The figure shows that after abrupt drug discontinuation the symptoms of insomnia (measured as total wake time) not only promptly return but there is a rebound of insomnia, that is worsening of the symptoms far above disease baseline from pre-treatment period (red arrow), in fact, this worsening is considerably greater than the maximal degree of improvement of sleep during the drug treatment. Adapted from Kales *et al.* (1983a).

The mechanisms that underlie the occurrence of the rebound effect are not yet fully elucidated; also, for the different CNS drugs the brain mechanism implicated in rebound may differ.

Generally, rebound is explained as a consequence of down-regulation and desensitization of the receptors targeted by the therapeutic drug, where increased severity of disease symptoms is related to the lag in time for receptors to be synthesized after the decrease in receptor sites during treatment (Fontaine *et al.*, 1984; Bhanji *et al.*, 2006). An alternative explanation is that there is a lag in production and replacement of endogenous neurotransmitters after their production was suppressed during treatment with the drug (Kales *et al.*, 1983b).

The treatment options for the rebound are the same as for acute withdrawal syndrome, but there is one more option, namely, the rebound symptoms also often improve rapidly after reintroduction of the discontinued drug, if it is not medically contraindicated (Chouinard and Chouinard, 2015; Chouinard *et al.*, 2017), then the drug should be tapered very slowly.

### Benzodiazepine rebound

One of the most common examples is anxiety and insomnia rebound following abrupt discontinuation of treatment with BZ. Symptoms usually appear within 1–5 days of drug discontinuation, depending on the half-life of the individual drug, and may last at least 3 days for insomnia rebound (Kales *et al.*, 1983a, 1987; Bixler *et al.*, 1985), and at least 2 weeks for anxiety rebound (Fontaine *et al.*, 1984), but frequently the rebound was not evaluated for all its full duration. Patients experienced rebound insomnia after treatment with several short- and intermediate-acting BZ used for insomnia, including alprazolam, lorazepam, nitrazepam, triazolam, midazolam, flunitrazepam and lormetazepam (Kales *et al.*, 1978, 1983a, 1987; Bixler *et al.*, 1985; Lader and Lawson, 1987; Roehrs *et al.*, 1992). Figure 5 shows rebound insomnia after abrupt withdrawal of treatment with triazolam (Kales *et al.*, 1983b). However, rebound insomnia was not reported following use of long-acting BZ such as quazepam and flurazepam (Kales *et al.*, 1982; Bixler *et al.*, 1985).

Rebound anxiety or panic attacks resulted from abrupt withdrawal of BZ such as alprazolam, lorazepam, diazepam, clorazepate and bromazepam (Scharf *et al.*, 1982; Fontaine *et al.*, 1984; Pecknold *et al.*, 1988; Rickels *et al.*, 1988; Roy-Byrne *et al.*, 1989; Chouinard, 2004). Anxiety patients more frequently experience rebound with short- and intermediate-actin BZ than long-acting (Chouinard, 2004).

Rebound insomnia was also reported for a non-BZ hypnotic drug, zolpidem (Voshaar *et al.*, 2004).

**Figure 6:**
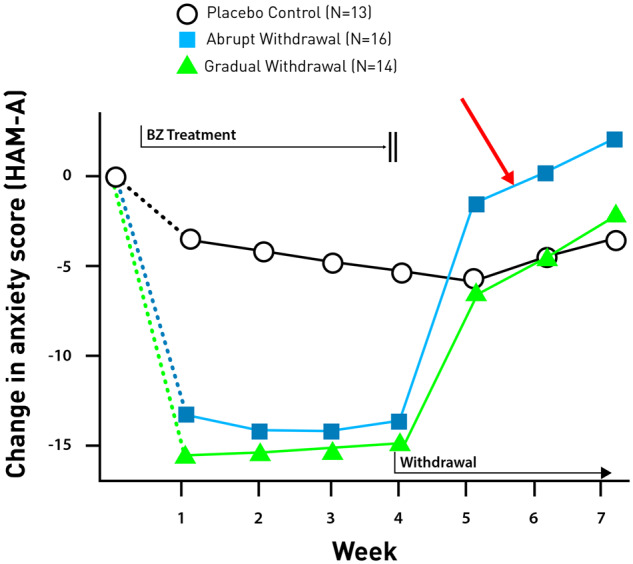
**Rebound anxiety in patients treated with diazepam or bromazepam who were abruptly withdrawn.** The figure shows anxiety scores (HAM-A) for three arms in the study: patients who were treated with BZ but then either discontinued drug abruptly (blue line) or discontinued drug gradually (green line), the study included also a placebo arm (black line). After abrupt drug discontinuation the symptoms of anxiety not only return to pre-treatment state, but there is also a rebound of anxiety (red arrow), that is a worsening of the symptoms above disease baseline. However, the gradual drug discontinuation does not produce the rebound and is similar to placebo arm. Adapted from Fontaine *et al.* (1984).

Following is an example of rebound anxiety after withdrawal of BZ in patients with generalized anxiety disorder treated successfully with diazepam or bromazepam (Fontaine *et al.*, 1984) and Fig. 6. The patients were treated for 4 weeks with BZ, then one group was abruptly withdrawn, and the second group gradually withdrawn. The scores of patients withdrawn abruptly increased 10% or more on both the Hamilton Rating Scale for Anxiety and the Self Rating Symptom Scale. In patients with rebound anxiety, previous anxiety symptoms returned and were more severe than before treatment, beginning in some cases within 24 h of drug withdrawal.

Also, Z-drugs such as zopiclone were reported to cause rebound insomnia (Soldatos *et al.*, 1999; Tsutsui and Zolipidem Study Group, 2001; Cimolai, 2007).

### Stimulant rebound

Stimulants such as methylphenidate and amphetamine may cause rebound in children with attention-deficit/hyperactivity disorder, which can include serious and consistent deterioration of behaviour manifested in sadness, crying, emotional lability, irritability, social withdrawal, restlessness, distractibility, belligerence, excitability, talkativeness, euphoria and insomnia, emerging after the last stimulant dose has worn off (Rapoport *et al.*, 1978; Porrino *et al.*, 1983; Smucker and Hedayat, 2001; Carlson and Kelly, 2003; Connor, 2015). In fact, up to one-third of children taking methylphenidate for attention-deficit/hyperactivity disorder experience a rebound as interdose recurrence of symptoms in the late afternoon when the medication wears off and symptoms of attention-deficit/hyperactivity disorder, such as irritability and non-compliance, return (Riccio *et al.*, 2001); however, the total duration is not known as the next dose of drug is given; in more severe cases the medication is discontinued.

### Selective serotonin reuptake inhibitor’s rebound

Rebound involves the return of symptoms of the treated disorder, but at greater intensity. These include: anxiety, panic, agitation, insomnia, depression, obsessions and compulsions (Bhanji *et al.*, 2006; Chouinard and Chouinard, 2015). Generally, peak of onset is 36–96 h which depends mainly on drug duration of action, whereas the duration can be up to 6 weeks, which depends on drug elimination half-life (Chouinard and Chouinard, 2015).

### Antipsychotic rebound

Rebound psychosis, was noted not only for many classic antipsychotics, such as haloperidol; but also, atypical antipsychotics such as quetiapine and clozapine. Rebound psychosis may include positive symptoms from pre-treatment level, however, at greater severity: illusions, hallucinations, delusions, catatonia, Schneider passivity experiences (Borison *et al.*, 1988; Kahne, 1989; Margolese *et al.*, 2002; Chouinard *et al.*, 2017). The peak of onset is 36–96 h after drug discontinuation or dose reduction and may last up to 6 weeks depending on drug elimination (Chouinard *et al.*, 2017).

### Immunomodulatory drug rebound

Recently, a new rebound syndrome was identified for immunomodulatory drugs indicated for treatment of multiple sclerosis, namely Gilenya (fingolimod) and natalizumab. The abrupt withdrawal of these drugs produces severe, sometimes life-threatening or fatal rebound in multiple sclerosis patients, accompanied by drastic increases in new lesions or enhancing lesions seen on MRI (Sorensen *et al.*, 2014; Faissner *et al.*, 2015; Hatcher *et al.*, 2016; Czlonkowska *et al.*, 2017; Gunduz *et al.*, 2017; Larochelle *et al.*, 2017).

### Summary points

- Rebound reflects drug’s ability to affect organism’s neuroadaptation expressed as tolerance and dependence which are exposed during drug discontinuation; thus, rebound is more severe for drugs which produce tolerance and dependence within a short time and to a higher degree.
- Rebound coincides in time with acute withdrawal syndrome but is differentiated from it by the fact that represents the symptom of the underlying disorder but of the greater severity than before the treatment, whereas acute withdrawal represents a new symptom.
- Rebound is of relatively short duration and self-terminating, although at times may be severe and life-threatening.

## Part IV. Protracted withdrawal syndrome—definition and examples

Protracted withdrawal also called post-acute withdrawal syndrome (PAWS), persistent post-withdrawal syndrome, and prolonged withdrawal syndrome, describes a set of persistent impairments that occur after withdrawal from some drug classes, such as BZ, opioids, antipsychotics and antidepressants. Symptoms of protracted withdrawal frequently appear later, well past the expected timeframe for acute withdrawal, last longer, and sometimes are irreversible. Because many names are used to describe protracted withdrawal symptoms, which makes identification confusing at times, we present a list of all known to date names of this syndrome, compiled by the Substance Abuse and Mental Health Services Administration (SAMHSA, 2010).

- Chronic withdrawal
- Extended withdrawal
- Late withdrawal
- Long-term withdrawal
- Persistent post-use symptoms
- Post-acute withdrawal syndrome
- Post-use syndrome
- Protracted abstinence
- Sobriety-based symptoms
- Subacute withdrawal

The most characteristic feature of this disorder is its duration, sometimes persisting for weeks and months after cessation of the drug; additionally, new disorders may emerge.

Protracted withdrawal syndrome is not only characteristic of drugs of abuse but also was described for non-scheduled drug classes, such as SSRIs and antipsychotics. In this study, we present some known examples of post-acute withdrawal syndromes.

### Scheduled drugs

#### Opioid protracted withdrawal syndrome

Protracted withdrawal symptoms related to opioid withdrawal include: opioid cravings, memory problems, inability to think clearly, fatigue, anxiety, depression, dysphoria, irritability, sleep disturbances, insomnia, palpitations, restlessness, periodic diarrhoea and hypomania (Kolb, 1928; Satel *et al.*, 1993; SAMHSA, 2010). Some symptoms, such as fatigue, insomnia and restlessness can last for weeks or months following withdrawal from opioids, in some cases, 6–9 months.

Some opioid drugs may produce physiological protracted withdrawal symptoms. During abrupt morphine withdrawal, Martin and Jasinski (1969) observed after the acute withdrawal phase occurrence of a protracted or secondary phase which usually began to emerge between the sixth and ninth weeks after drug withdrawal and persisted through the weeks 26–30. This phase was characterized by decreased blood pressure, heart rate and body temperature, as well as miosis and hyposensitivity of the respiratory centre to CO_2._

#### Benzodiazepine protracted withdrawal syndrome

Protracted withdrawal symptoms include: anxiety, depression, psychotic reactions, memory impairment, motor symptoms (muscle jerking, blepharospasm), paraesthesia, formication, tinnitus and irritable bowel syndrome, and there is a characteristic fluctuation of symptoms that may wax and wane (Smith and Wesson, 1983; Ashton, 1991; SAMHSA, 2010). Generally, the symptoms of protracted withdrawal start ∼4–6 weeks after drug discontinuation and may last 6–12 months, but some symptoms, such as anxiety, may persist for up to 2 years (Ashton, 1991).

#### Stimulants protracted withdrawal

Protracted withdrawal sometimes called the extinction phase is characterized by symptoms such as fluctuations in mood and energy levels, irritability, restlessness, anxiety, agitation, fatigue, lacking energy and anhedonia, cravings and disturbed sleep and this phase may last for weeks to months (New South Wales Government, 2008).

### Non-scheduled drugs

#### SSRIs and SNRIs protracted withdrawal syndrome

Protracted withdrawal symptoms include: tardive insomnia, major depression, bipolar disorder, panic disorder, agitation, anxiety disorders and panic attacks, emotional lability, mood swings, irritability, aggression, impaired concentration, impaired memory and pathological gambling. These disorders can last for several months to years when the previous drug treatment is not restarted (Bhanji *et al.*, 2006; Belaise *et al.*, 2012, 2014; Chouinard and Chouinard, 2015).

#### Antipsychotic protracted withdrawal syndrome

Main protracted withdrawal symptoms for antipsychotics include tardive dyskinesia and supersensitivity psychosis (Crane, 1968; Chouinard and Jones, 1980; Margolese *et al.*, 2005; Chouinard and Chouinard, 2008; Oda *et al.*, 2015; Chouinard *et al.*, 2017; Nakata *et al.*, 2017). The peak of onset is 24 h to 6 weeks after drug discontinuation or dose reduction or switch and protracted withdrawal may last several months (Chouinard *et al.*, 2017).

### Summary points

- Protracted withdrawal is a relatively less known aspect of dependence and withdrawal; however, its duration and severity affect the ability of subjects to terminate therapeutic drugs and drugs of abuse and addiction.
- Protracted withdrawal may represent very slowly reversible or permanent pathological changes in the neurocircuitry.

## Part V: Evaluation of drug dependence in clinical studies—regulatory considerations

### Human dependence study—design and considerations

Evaluation of dependence, withdrawal and rebound for new drugs being developed is of great importance, as it provides information related to potential drug scheduling and safe use. The evaluation provides critically important safety information for patients and physicians, especially in cases when the drug must be abruptly withdrawn due to serious, life-threatening adverse events.

It is probably important to note here that for regulatory purposes an animal dependence study is not an alternative to a human dependence evaluation. It is only a first step, one which is important for gathering information about potentially serious adverse events of withdrawal, such as convulsions.

The recommended design of a clinical dependence study contains consideration of the following components: population, design, statistical considerations, dose, pharmacodynamic and pharmacokinetic assessments, evaluation of rebound, adverse events collection and analysis. All listed technical details and considerations are available as the Supplementary material 1.

## Discussion

Major issues in the evaluation of dependence and withdrawal involve the definitions and nomenclature currently used. Both can possibly contribute to misunderstanding and confusion. This particularly affects different authors’ naming conventions for acute withdrawal syndrome, and even more so, for protracted withdrawal syndrome.

In general, the authors consider the nomenclature of withdrawal syndrome and the time course of withdrawal phenomena, presented by Chouinard and Chouinard (2015) to be clear and comprehensive. This nomenclature and the presentation of the different aspects of withdrawal phenomena could be adapted to describe withdrawal from other drugs and drug classes.

It is important to note that some drugs and drug classes were not mentioned in this short review. This does not necessarily mean that these drugs do not produce a withdrawal syndrome, it may simply mean that withdrawal has never been evaluated and described.

## Conclusions

As discussed above, the evaluation of dependence and withdrawal is critically important for CNS-active drugs, which are likely to be scheduled. However, the withdrawal evaluation is also important for non-scheduled CNS-active drugs, mainly for safety reasons. Reidenberg (2011) summarizing issues related to drug dependence and withdrawal, stated: ‘Drug discontinuation effects can occur and are usually neglected in pharmacology and medicine until adverse clinical events force them to be noticed’. It is vital that dependence, withdrawal and rebound be recognized and understood by healthcare practitioners, because these effects may constitute major safety issues. The data on acute withdrawal symptoms and rebound obtained from the study and evaluation of dependence will provide physicians and patients with information on drug tapering and constitute a warning about possible effects related to abrupt drug discontinuation (Kosten and O’Connor, 2003).

Except for a few drug groups comprising highly addictive drugs, such as opioids, BZ and stimulants; and non-scheduled drugs such as SSRIs, there is relatively little knowledge of different aspects of the withdrawal phenomena for most currently used drugs. But even for these drug classes mentioned above, product labels often do not always reflect all current information available in the scientific literature and fail to provide adequate warnings for patients and physicians.

The evaluation of dependence and withdrawal for drugs in paediatric population is very important and should be also addressed in the future. The current available data are more than sparse and only available for some opioid and BZ drugs and some stimulants. Also, the sex differences in the withdrawal symptomatology should be further explored as the data for many scheduled and unscheduled drug is not available.

This is the authors’ hope that future drug development will incorporate many of the methods described in this review as the knowledge of the withdrawal symptoms is critical for patients’ safety. Also, equally important is incorporating knowledge on withdrawal and dependence in the drug labels.

## Supplementary Material

fcz025_Supplementary_Data

## Abbreviations

ARSW: Adjective Rating Scale for Withdrawal
ASAM: American Society of Addiction Medicine
BZ: benzodiazepines
CB1: cannabinoid 1 receptors
COWS: Clinical Opiate Withdrawal Scale
DSM-5: diagnostic and statistical manual of mental disorders, fifth edition
FDA: Food and Drug Administration
INF: information not found
MAOIs: monoamine oxidase inhibitors
NMS: neuroleptic malignant syndrome
NAS: neonatal abstinence syndrome
SSRIs: selective serotonin reuptake inhibitors
SNRIs: serotonin–norepinephrine reuptake inhibitors
THC: Δ9-tetrahydrocannabinol
VAS: Visual Analogue Scale
