# Supplementary material for: Dependence, withdrawal and rebound of CNS drugs: an update and regulatory considerations for new drugs development
Source: Brain Commun. 2019 Oct 16;1(1):fcz025. doi: 10.1093/braincomms/fcz025 (PMC7425303; doi:10.1093/braincomms/fcz025)
Supplement: fcz025_Supplementary_Data [file fcz025_supplementary_data.zip › Supplementary material # 1. Design of human dependence evaluation Oct 24 2019 Version 3.docx]

**Supplementary material # 1**

- - 1. **Design of human dependence evaluation or study**
- **Population**

The study should be conducted in patients for whom the drug will be prescribed. In such a population, all important aspects of withdrawal can be evaluated, such as new emerging acute withdrawal symptoms, rebound, and even sometimes protracted withdrawal, all of which affect drug safety. Sometimes, the symptom of craving can be assessed as well, especially for drug groups which may be abused and for which withdrawal scales contain questions about craving; for example, the Amphetamine Withdrawal Questionnaire (AWQ) or Marijuana Withdrawal Checklist (MWC).

At times, the evaluation of dependence in the patient population may not be possible. There are instances in which abrupt discontinuation of a drug is medically contraindicated in patients’ population due to known serious adverse events, such as seizure in epilepsy patients or worsening of psychiatric disorders in patients treated for psychosis or depression. In such cases, withdrawal information can be obtained from patients who were withdrawn from the clinical studies for various reasons. Also, this information can be obtained during the post-marketing period as a part of enhanced pharmacovigilance for withdrawal adverse events with quarterly updates, for at least 3 years. A dependence study in healthy subjects is not recommended.

- **Design**

The dependence study should preferably be conducted at the end of the clinical trial Phase II or III, for at least four-five weeks. If it is performed as an independent dependence study in patients, the maintenance phase should last at least four-five weeks, as it is the shortest time of dependence formation known to authors (Hollister *et al.*, 1963; MacKinnon and Parker, 1982) and which would assure at least three-four weeks of a drug’s steady state. The study should include two arms: active drug arm and a placebo arm. During the maintenance phase, both drug and placebo arms should be double blind and stay double blind or at least single blind throughout the withdrawal period. This is done to blind the patients for the potential effects of expected drug withdrawal.

The discontinuation period should last at least 4-5 weeks but might be longer for drugs with very long half-lives. After the drug discontinuation, patients should have follow-up visits for pharmacodynamic (PD) and pharmacokinetic (PK) assessments for at least four-five weeks at predetermined time points; usually the visits are more frequent during the first week of withdrawal ~2-3 times a week and then at least weekly for 4 weeks, then phone calls are helpful for up to six weeks, to check for the presence of protracted withdrawal. Based on clinical and nonclinical data, additional safety precautions may need to be included during this phase to address unknown adverse events that may occur.

The main study endpoints include the primary endpoint which is a withdrawal questionnaire and 2-3 secondary endpoints which usually include one of the additional scales (depression, anxiety, sleep) and withdrawal adverse events.

- **Statistical considerations**

The number of subjects who completed the study should be adequate to show statistical significance of the study based on primary and secondary endpoints considerations. There should be adequate gender representation, especially if other nonclinical and clinical data indicate sex differences for adverse events.

- **Dose**

The dose used during the maintenance phase should be the highest therapeutic dose. If a dependence study is performed at the conclusion of a clinical trial in which patients were given different doses, the number of patients using the maximal dose should be substantial (or statistically appropriate). The dose should not be changed during the maintenance phase (drug administration period) unless some subjects do not tolerate the highest dose, but do tolerate lower doses.

- **Pharmacodynamic (PD) assessments**

During the maintenance phase, appropriate PD scales and withdrawal questionnaires specific to a drug class should be administered at baseline (pre-treatment) and on the last day of drug administration (withdrawal baseline). During the withdrawal phase, PD scales and questionnaires should be administered as follows: on the first day of the abrupt discontinuation of the drug, then according to half-life and possible formation of active metabolites, but usually more frequently during the first week; on days 2-3-5, then usually twice a week or even weekly.

Sometimes evaluation of dependence and withdrawal syndromes for new molecular entities (NME) may be quite challenging, as more than one neurotransmitter system is affected based on nonclinical studies. In such cases, use of one or more withdrawal scales is generally recommended; choice of the scales is based on the adverse events profile during the clinical trials as well as any other relevant information from nonclinical and in vitro studies. On occasion, based on this data, it may be necessary to use withdrawal questionnaires for two or even three different pharmacological groups.

Several withdrawal scales were developed for specific drug classes:

*Opiate withdrawal scales*

- Clinical Opiate Withdrawal Scale (COWS)
- Subjective Opiate Withdrawal Scale (SOWS)

*Benzodiazepine withdrawal scales*:

- Physicians Withdrawal Checklist PWC-20 and PWC-34
- Tyrer’s Benzodiazepine Withdrawal Symptom Questionnaire (BWSQ)
- Benzodiazepine Dependence Questionnaire (BDEPQ)
- Clinical Institute Assessment of Withdrawal-Benzodiazepines (CIAW-B)

*Stimulant withdrawal scales:*

- Amphetamine Withdrawal Questionnaire (AWQ)
- Cocaine Selective Severity Assessment (CSSA)

*Cannabinoid withdrawal scales*:

- Cannabis Withdrawal Scale (CWS)
- Marijuana Withdrawal Checklist (MWC)

*SSRI withdrawal scale****:***

- Discontinuation Emergent Signs and Symptoms Checklist (DESS)

There are also pediatric withdrawal scales that measure benzodiazepines and opioids withdrawal:

- Sophia Observation Withdrawal Symptoms-scale (SOS)
- Withdrawal Assessment Tool-1 (WAT-1)

Additional scales and measures can be helpful in evaluating aspects of withdrawal. It is also recommended that only validated scales be used.

*Depression, suicidality and mood scales*

- Columbia-Suicide Severity Rating Scale (C-SSRS)
- Profile of Mood State - Bipolar (POMS-Bi)
- Depression Scales:
  - Hamilton Depression Rating Scale (HDRS)
  - Montgomery-Asberg Depression Rating Scale (MADRS)
  - Beck Depression Inventory
  - Hospital Anxiety and Depression Scale (HADS)

*Anxiety Scales*

- Hamilton Anxiety Rating Scale (HAM-A)
- Spielberger State Anxiety Inventory (SSAI) Short form

*Sleep scales*

- Pittsburgh Sleep Quality Index (PSQI)
- Epworth Sleepiness Scale (ESS)

*Cognitive scales*

- Hopkins Verbal Learning Test – Revised (HVLT-R)
- Divided Attention Test (DAT)
- Digit-Symbol Substitution Task (DSST)

*Subject-rated Visual Analogue Scales (VAS)*

- Anxiety VAS
- Sick VAS
- Pain VAS
- Nausea VAS

Sometimes it is also important to obtain physiological measures, such as vital signs, as the autonomic nervous system is almost always affected during withdrawal. Certain measures are drug-specific, such as pupil size during opioid withdrawal or bradycardia during stimulants withdrawal:

- Pupil diameter
- Respiratory rate (RR)
- Arterial oxygen saturation
- Skin temperature
- Systolic and diastolic blood pressure (SBP and DBP)
- Heart rate (HR)
- **Evaluation of rebound phenomena**

Because the dependence study is conducted in the patient population, evaluation of the rebound phenomena is recommended during the withdrawal period. Rebound is an aspect of drug withdrawal and provides prescribers and patients with important safety information. To assess rebound, neurological and psychiatric scales used to assess drug efficacy during Phase II and III trials should continue to be administered during the whole withdrawal period.

*Examples of scales for Neurological disorders:*

- Unified Parkinson’s Disease Rating Scale (UPDRS)
- Unified Huntington’s Disease Rating Scale (UHDRS)
- Yale Global Tic Severity Scale (YGTSS)
- The Essential Tremor Rating Assessment Scale (TETRAS)
- Abnormal Involuntary Movement Scale (AIMS)
- Berg Balance Test Score (BBT)
- Barnes Akathisia Rating Scale (BARS)
- Sleep scales (as above)

*Examples of scales for Psychiatric Disorders:*

- Depression scales (as above)
- Columbia-Suicide Severity Rating Scale (C-SSRS)
- ADHD scales (examples)
  - Conners Adult ADHD Rating Scale Screening Version (CAARS)
  - Attention Deficit Hyperactivity Disorder-Rating Scale IV (ADHD-RS-IV)
- Schizophrenia, psychosis scales (examples)
  - Positive and Negative Syndrome Scale for Schizophrenia (PANSS)
  - Brief Psychiatric Rating Scale (BPRS)
- Anxiety scales (as above)
- **Blood sampling - for PD-PK correlation**:

Blood sampling should be considered only for the independent dependence studies. Time points for blood sampling should follow the time points for pharmacodynamic (PD) assessments. The rationale for PK evaluation is to help to distinguish between adverse events due to withdrawal from symptoms of drug toxicity. These symptoms may sometimes be identical, so, PK is critical to provide the clarification. For example, nausea and headache are some of the most common AEs due to drug toxicity and seen during the clinical trials, but they are also very common withdrawal symptoms.

- **Adverse events** **(AEs)** **collection**

Adverse events should be reported separately for the treatment and withdrawal phases and preferably be organized into smaller time periods for the withdrawal phase: first week, second week, etc. Further classification of AEs by gender and age (children, adults, older patients) if available, should provide additional information on the specific withdrawal characteristics.

- **Analysis of the data**

Analysis of the various scales and assessments should be performed for the specified *a priori* endpoints. There are primary and secondary endpoints in the analysis. The primary endpoint generally includes the main withdrawal questionnaire, and secondary endpoints include withdrawal AEs and maybe one or more other scales. If there is evidence of withdrawal the analysis should be performed for the specified *a priori* time points. This is to assure that critical time points of potential withdrawal syndromes will be captured, including onset, peak, duration and extinction of withdrawal, and possibly onset and severity of rebound phenomena.

If different drug doses were used during the clinical trial, all analyses for the withdrawal period should be also performed for all doses separately to find out if there is dose effect that influences severity of withdrawal symptoms.

In addition to a descriptive summary, all data should be presented in tables and figures.

- ***Analysis of acute withdrawal syndrome***

Evaluation of the symptoms of acute withdrawal will consist of the following:

1. Adverse events that occurred during the withdrawal period of 4-6 weeks after drug discontinuation
2. Scores of the drug-specific withdrawal and accessory scales
3. Changes in vital signs during the withdrawal period

- ***Analysis of rebound effect***

The rebound effect is evaluated during the assessment of acute withdrawal after drug discontinuation. An increase of the disease scores above the baseline (the pre-treatment period) level measured by disease-specific scales indicates rebound. Examples are presented in Figures 4 and 5.

- ***Analysis of protracted withdrawal syndrome***

Due to the usually later occurrence of protracted withdrawal symptoms, it may be difficult to capture the syndrome during the 4-6 weeks of acute withdrawal assessment. However, appearance of new symptoms during the late stages of acute withdrawal, and increasing intensity of some withdrawal symptoms, may potentially indicate development of protracted withdrawal. To evaluate for potential protracted withdrawal, it is recommended that patients have long-term follow-ups to collect all adverse events which occur after acute withdrawal syndrome; a follow-up period of at least 2-3 months is suggested. Post-marketing monitoring for protracted withdrawal is particularly indicated for drugs affecting dopaminergic, serotonergic and GABA-ergic systems. Protracted withdrawal syndromes were described for drugs affecting these systems.

The drug developers should plan to conduct clinical dependence studies early in development, even pre-IND (Investigational New Drug Application), and discuss the need to implement evaluation of dependence and withdrawal studies during Phase II and Phase III clinical studies prior to their initiation. Also, the collection of withdrawal AEs from all clinical studies is still required, and they can easily be obtained during follow-up visits.

1. **Limitations of clinical dependence evaluation**

Clinical dependence study/evaluation has several limitations.

- **Time factor**

The first and the most important is the time of exposure to the drug. If the dependence evaluation is performed at the conclusion of clinical trials in Phase II and III lasting for many months, the time factor is not an issue. However, if the dependence study is performed as an independent study with a maintenance phase lasting approximately four-five weeks, the duration of exposure might be too short for some drugs (and possible active metabolites) to develop dependence and manifest the full spectrum of withdrawal syndrome. Also, the study’s follow-up period, usually 4-5 weeks, might be too short to capture protracted withdrawal syndromes.

- **Population**

The main concern generally is that the number of patients enrolled in the dependence evaluation and providing the full sets of requested data is frequently relatively small, therefore might be too small to be representative of the patient population. Thus, it is recommended that at least the full data sets from 100 patients be provided.

- **Regulatory considerations**

Finally, there are also a number of regulatory steps that could improve planning and collection of the data necessary to evaluate dependence and withdrawal of new drugs which should include:

1) Nonclinical dependence study in a species with similar metabolism to humans

2) Clinical dependence evaluation/study

3) Collection of withdrawal AEs from clinical trials obtained at follow-up visits

4) Planning a clinical dependence study early in development, even at the pre-IND stage

5) Obtaining input from the FDA on implementing evaluation of dependence and withdrawal during Phase II and Phase III clinical studies; as mentioned earlier it is preferable that dependence and withdrawal be evaluated at the end of Phase II or III in patient populations when the maximal therapeutic dose is known.
