# Supplementary material for: Dependence, withdrawal and rebound of CNS drugs: an update and regulatory considerations for new drugs development
Source: Brain Commun. 2019 Oct 16;1(1):fcz025. doi: 10.1093/braincomms/fcz025 (PMC7425303; doi:10.1093/braincomms/fcz025)
Supplement: fcz025_Supplementary_Data [file fcz025_supplementary_data.zip › Supplemental Material # 2-Withdrawal syndromes Sep 26 2019.pdf]

Supplemental Material # 2

Less frequent acute withdrawal syndromes.

Monoamine Oxidase Inhibitors Withdrawal Syndrome

Some more frequently used MAO inhibitors include phenelzine, selegiline, rasagiline and tranylcypromine. Discontinuation of MAO inhibitors which are still used as antidepressants and anxiolytics, such as phenelzine and tranylcypromine, can result in palpitations, nausea, vomiting, diarrhea, hyperphagia, weakness, sweating, restlessness, irritability, agitation, aggression, pressured speech, insomnia, somnolence, nightmares, myoclonic jerks, clonus, ataxia, athetosis, anxiety, paresthesia, such as electric shock sensations, visual, olfactory and tactile hallucinations, cognitive impairment, disorientation, depersonalization, labile affect, hypomania, acute psychosis, paranoid delusions, delirium, aggressiveness, catatonia, convulsions, and suicidality (Liskin *et al.*, 1985; Dilsaver, 1990; Halle and Dilsaver, 1993; Dilsaver, 1994) and labels e.g., Parnate 2018; Nardil 2007).

Gabapentin Withdrawal Syndrome (drug scheduled now in Kentucky, Tennessee, West Virginia, Michigan**)**

Gabapentin withdrawal syndrome was described only very recently and may include agitation, confusion, disorientation, anxiety, tremor, gastrointestinal distress, diaphoresis, tachycardia or palpitations, insomnia, somatic pain, flu-like symptoms with chills, occasional hypertension, asterixis and myoclonus; catatonia, status epilepticus, delusions, and hallucinations (Norton, 2001; Mah and Hart, 2013; Mersfelder and Nichols, 2016). Withdrawal usually occurs within 12 hours to seven days of drug discontinuation, and with most cases occurring within the first two days.

Beta-blocker Withdrawal Syndrome

Some more frequently used beta-blockers include propranolol, atenolol, metoprolol and pindolol. The symptoms of withdrawal include nervousness, restlessness, anxiety, palpitations, abdominal cramps and pain, anorexia, nausea, vomiting, insomnia, headaches, vivid dreams, malaise, fatigue, tremor, diaphoresis, tachycardia, excessive salivation and cardiovascular morbidity (Houston and Hodge, 1988). However, the symptoms of withdrawal frequently also depend on the treated patient

population. In patients treated for angina pectoris with beta-blockers, withdrawal may cause new arrhythmias, including ventricular tachycardia, hypertension, myocardial infarction, and even death; however, some of these phenomena likely may represent a rebound (Slome, 1973; Alderman *et al.*, 1974; Miller *et al.*, 1975; Frishman, 1987).

Statin Withdrawal Syndrome

Some more frequently used statins include simvastatin, atorvastatin and fluvastatin.

In 1998, Thomas et al. (Thomas and Mann, 1998) observed that patients who were switched from simvastatin (which was more expensive), to another statin fluvastatin (which was fully subsidized to be made available), at less than the equivalent dose of the statin used previously (simvastatin) had a tripling of their cardiovascular event rate over the next six months. The events included myocardial infarctions, unstable anginas, and non-hemorrhagic strokes. Statin withdrawals or dose decreases may increase levels of low-density lipoproteins (LDL) and the frequency of thrombotic events. In patients with acute coronary syndromes, statin withdrawal was associated with an increased risk of cardiovascular events; and in the patients with non-ST-elevation myocardial infarction who discontinued statins during hospitalization, rates of chronic heart failure, pulmonary edema, arrhythmia, cardiac arrest, cardiogenic shock, and death increased compared with those of patients continuing statin therapy (Heeschen *et al.*, 2002; Spencer *et al.*, 2004; Cubeddu and Seamon, 2006). However, in patients with stable cardiac conditions, a short-term discontinuation of statins does not seem to increase the risk for an acute coronary event (McGowan and Treating to New Target Study, 2004). In patients with ischemic stroke, stopping of statins for three days was associated with an increased risk of death, greater neurological deterioration, and a larger infarction volume (Blanco *et al.*, 2007).

Corticosteroid Withdrawal Syndrome

Frequently used corticosteroids include cortisol, prednisone and dexamethasone. Withdrawal symptoms are characterized by fever, anorexia, nausea, weight loss, fatigue, myalgias, arthralgias, headache, lethargy, restlessness, fever and skin desquamation of face, hands and feet. Less frequently seen are: abdominal pain, vomiting, postural hypotension, hyponatremia and hyperkalemia (Henneman *et al.*, 1955; Nicolaides *et al.*, 2000; Hochberg *et al.*, 2003; Margolin *et al.*, 2007). However, serious adverse events may occur, such as mania, delirium and psychosis

with catatonia (Judd *et al.*, 1983; Nicolaides *et al.*, 2000) also suppression of hypothalamic-pituitary-adrenocortical (HPA) function, which may lead occasionally to coma or fatal postoperative shock related to atrophy of the adrenal cortex (Fraser *et al.*, 1952; Silove, 1962). Generally, withdrawal symptoms begin to appear from 24 to 48 hours after the last dose, and usually resolve after several days (Henneman *et al.*, 1955).

Aspirin Withdrawal Syndrome

One of the lesser-known withdrawal syndromes is that appearing after chronic aspirin use (Reidenberg, 2011). Studies have shown that abrupt discontinuation of aspirin after taking it chronically may increase the risk of having a heart attack or stroke (Harvard Health Letter, 2005). Aspirin withdrawal in patients with coronary artery disease may present a serious risk for the occurrence of a new coronary event which may occur in approximately 10 days from aspirin withdrawal (Ferrari *et al.*, 2005). Also, discontinuation of low-dose aspirin prescribed for the secondary prevention of cardiovascular or cerebrovascular events increased the risk of ischemic stroke and transient ischemic attack (TIA) (Garcia Rodriguez *et al.*, 2011). This effect on the increase of early thromboembolic complications in patients treated with aspirin for prevention of ischemic vascular disease is considered by some a rebound in platelet activity following aspirin withdrawal (Lordkipanidze *et al.*, 2009).

References

Alderman EL, Coltart DJ, Wettach GE, Harrison DC. Coronary artery syndromes after sudden propranolol withdrawal. Ann Intern Med 1974; 81(5): 625-7.

Blanco M, Nombela F, Castellanos M, Rodriguez-Yanez M, Garcia-Gil M, Leira R, *et al.* Statin treatment withdrawal in ischemic stroke: a controlled randomized study. Neurology 2007; 69(9): 904-10.

Cubeddu LX, Seamon MJ. Statin withdrawal: clinical implications and molecular mechanisms. Pharmacotherapy 2006; 26(9): 1288-96.

Dilsaver SC. Heterocyclic antidepressant, monoamine oxidase inhibitor and neuroleptic withdrawal phenomena. Prog Neuropsychopharmacol Biol Psychiatry 1990; 14(2): 137-61.

Dilsaver SC. Withdrawal phenomena associated with antidepressant and antipsychotic agents. Drug Saf 1994; 10(2): 103-14.

Ferrari E, Benhamou M, Cerboni P, Marcel B. Coronary syndromes following aspirin withdrawal: a special risk for late stent thrombosis. J Am Coll Cardiol 2005; 45(3): 456-9.

Fraser CG, Preuss FS, Bigford WD. Adrenal atrophy and irreversible shock associated with cortisone therapy. J Am Med Assoc 1952; 149(17): 1542-3.

Frishman WH. Beta-adrenergic blocker withdrawal. Am J Cardiol 1987; 59(13): 26F-32F.

Garcia Rodriguez LA, Cea Soriano L, Hill C, Johansson S. Increased risk of stroke after discontinuation of acetylsalicylic acid: a UK primary care study. Neurology 2011; 76(8): 740-6.

Halle MT, Dilsaver SC. Tranylcypromine withdrawal phenomena. J Psychiatry Neurosci 1993; 18(1): 49-50.

Harvard Health Letter. Aspirin: Quitting cold turkey could be dangerous. 2005.

Heeschen C, Hamm CW, Laufs U, Snapinn S, Bohm M, White HD, *et al.* Withdrawal of statins increases event rates in patients with acute coronary syndromes. Circulation 2002; 105(12): 1446-52.

Henneman PH, Wang DM, Irwin JW, Burrage WS. Syndrome following abrupt cessation of prolonged cortisone therapy. J Am Med Assoc 1955; 158(5): 384-6.

Hochberg Z, Pacak K, Chrousos GP. Endocrine withdrawal syndromes. Endocr Rev 2003; 24(4): 523-38.

Houston MC, Hodge R. Beta-adrenergic blocker withdrawal syndromes in hypertension and other cardiovascular diseases. Am Heart J 1988; 116(2 Pt 1): 515-23.

Judd FK, Burrows GD, Norman TR. Psychosis after withdrawal of steroid therapy. Med J Aust 1983; 2(7): 350-1.

Liskin B, Roose SP, Walsh BT, Jackson WK. Acute psychosis following phenelzine discontinuation. J Clin Psychopharmacol 1985; 5(1): 46-7.

Lordkipanidze M, Diodati JG, Pharand C. Possibility of a rebound phenomenon following antiplatelet therapy withdrawal: a look at the clinical and pharmacological evidence. Pharmacol Ther 2009; 123(2): 178-86.

Mah L, Hart M. Gabapentin withdrawal: case report in an older adult and review of the literature. J Am Geriatr Soc 2013; 61(9): 1635-7.

Margolin L, Cope DK, Bakst-Sisser R, Greenspan J. The steroid withdrawal syndrome: a review of the implications, etiology, and treatments. J Pain Symptom Manage 2007; 33(2): 224-8.

McGowan MP, Treating to New Target Study G. There is no evidence for an increase in acute coronary syndromes after short-term abrupt discontinuation of statins in stable cardiac patients. Circulation 2004; 110(16): 2333-5.

Mersfelder TL, Nichols WH. Gabapentin: Abuse, Dependence, and Withdrawal. Ann Pharmacother 2016; 50(3): 229-33.

Miller RR, Olson HG, Amsterdam EA, Mason DT. Propranolol-withdrawal rebound phenomenon. Exacerbation of coronary events after abrupt cessation of antianginal therapy. N Engl J Med 1975; 293(9): 416-8.

Nicolaides NC, Pavlaki AN, Maria Alexandra MA, Chrousos GP. Glucocorticoid Therapy and Adrenal Suppression. Endotext 2000.

Norton JW. Gabapentin withdrawal syndrome. Clin Neuropharmacol 2001; 24(4): 245-6.

Reidenberg MM. Drug discontinuation effects are part of the pharmacology of a drug. J Pharmacol Exp Ther 2011; 339(2): 324-8.

Silove ED. Coma as presenting sign of adrenal failure after steroid withdrawal. A report of three cases. Br Med J 1962; 1(5277): 515-8.

Slome R. Withdrawal of propranolol and myocardial infarction. Lancet 1973; 1(7795): 156.

Spencer FA, Fonarow GC, Frederick PD, Wright RS, Every N, Goldberg RJ, *et al.* Early withdrawal of statin therapy in patients with non-ST-segment elevation myocardial

infarction: national registry of myocardial infarction. Arch Intern Med 2004; 164(19): 2162-8.

Thomas M, Mann J. Increased thrombotic vascular events after change of statin. Lancet 1998; 352(9143): 1830-1.
